# Extensive divergence of projections to the forebrain from neurons in the paraventricular nucleus of the thalamus

**DOI:** 10.1007/s00429-021-02289-6

**Published:** 2021-05-25

**Authors:** Sa Li, Xinwen Dong, Gilbert J. Kirouac

**Affiliations:** 1 Department of Oral Biology, Dr. Gerald Niznick College of Dentistry, Rady Faculty of Health Sciences, University of Manitoba, 780 Bannatyne Avenue, Winnipeg, MB R3E 0W2 Canada; 2 Institute of Psychology, Key Laboratory of Mental Health, Chinese Academy of Sciences, Beijing, 100101 China

**Keywords:** Stress, Paraventricular, Thalamus, Collaterals, Nucleus accumbens, Extended amygdala, Behavior

## Abstract

Neurons in the paraventricular nucleus of the thalamus (PVT) respond to emotionally salient events and project densely to subcortical regions known to mediate adaptive behavioral responses. The areas of the forebrain most densely innervated by the PVT include striatal-like subcortical regions that consist of the shell of the nucleus accumbens (NAcSh), the dorsolateral region of the bed nucleus of the stria terminalis (BSTDL) and the lateral-capsular division of the central nucleus of the amygdala (CeL). A recent tracing experiment demonstrated that the PVT is composed of two intermixed populations of neurons that primarily project to either the dorsomedial (dmNAcSh) or ventromedial region of the NAcSh (vmNAcSh) with many of the vmNAcSh projecting neurons providing collateral innervation of the BSTDL and CeL. The present study used triple injections of the retrograde tracer cholera toxin B to provide a detailed map of the location of PVT neurons that provide collaterals to the vmNAcSh, BSTDL and CeL. These neurons were intermixed throughout the PVT and did not form uniquely localized subpopulations. An intersectional viral anterograde tracing approach was used to demonstrate that regardless of its presumed target of innervation (dmNAcSh, vmNAcSh, BSTDL, or CeL), most neurons in the PVT provide collateral innervation to a common set of forebrain regions. The paper shows that PVT-dmNAcSh projecting neurons provide the most divergent projection system and that these neurons express the immediate early gene product cFos following an aversive incident. We propose that the PVT may regulate a broad range of responses to physiological and psychological challenges by simultaneously influencing functionally diverse regions of the forebrain that include the cortex, striatal-like regions in the basal forebrain and a number of hypothalamic nuclei.

## Introduction

The paraventricular nucleus of the thalamus (PVT) is one of the midline and intralaminar nuclei that was initially considered to be the final thalamic-cortical relay for the reticular activating system (Moruzzi and Magoun 1949; Groenewegen and Berendse 1994). Results from anatomical studies led to the refinement of this hypothesis by proposing that individual members of this group of thalamic nuclei were associated with specialized cortical-subcortical circuits (Alexander et al. 1990; Groenewegen and Berendse 1994). Interest in the PVT has noticeably increased with the recognition that this nucleus sends dense projections to subcortical regions important for the regulation of emotional and motivational behavior (details of the anatomy of the PVT are reviewed in Kirouac 2015, 2021). The areas of the forebrain most densely innervated by the PVT include the shell of the nucleus accumbens (NAcSh), the dorsolateral region of the bed nucleus of the stria terminalis (BSTDL) and the lateral-capsular division of the central nucleus of the amygdala (CeL) (Dong et al. 2017; Vertes and Hoover 2008; Li and Kirouac 2008). As a group, these striatal-like subcortical areas are conceptualized as a descending macrosystem involved in the regulation of adaptive behavior (Zahm 2006; Swanson 2000; Cardinal et al. 2002).

The PVT has been consistently identified as being activated by states of behavioral arousal and stress (Kirouac 2015; Hsu et al. 2014; Millan et al. 2017). Moreover, recordings of calcium signals or single unit activity in the PVT of awake animals exposed to unconditioned or conditioned stimuli with appetitive or aversive outcomes indicate that PVT neurons track the saliency of stimuli and promote behavioral responses to changing conditions (Zhu et al. 2018; Choi and McNally 2017). A considerable amount of direct experimental evidence is now available demonstrating that the PVT contributes to both appetitive and aversive responses in a projection-specific manner (Choi et al. 2019; Choi and McNally 2017; Zhu et al. 2016, 2018; Do-Monte et al. 2015, 2017; Labouebe et al. 2016; Cheng et al. 2018; Dong et al. 2020). For example, PVT neurons that project to the CeL were found to mediate the short-term anxiogenic effects of footshocks (Pliota et al. 2018) and the freezing associated with fear (Penzo et al. 2015; Do-Monte et al. 2015). This has led to an implicit view that a subpopulation of projection-specific neurons in the PVT engages the critical microcircuits in the CeL that mediate fear. Similar projection-specific effects have been described for PVT neurons that innervate the NAcSh with some groups reporting that this projection contributes to conditioned sucrose seeking (Labouebe et al. 2016; Cheng et al. 2018) while others reporting that activation of this projection reduces operant responses to a food reward (Lafferty et al. 2020; Do-Monte et al. 2017) or contributes to conditioned place avoidance (Zhu et al. 2016). It is possible that the inconsistent effects of modulating PVT afferents to the NAcSh reported in the literature may have been the result of experimental manipulations that differentially affected PVT fibers that preferentially innervate subregions of the NAcSh. For example, the dorsomedial region of the NAcSh (dmNAcSh) may have a role in mediating appetitive responses whereas the ventromedial NAcSh (vmNAcSh) may mediate aversive ones (Berridge and Kringelbach 2015; Castro and Berridge 2014; Al-Hasani et al. 2015). Consequently, activation or inhibition of PVT afferent inputs to these general regions of the NAcSh may have produced inconsistent or mixed effects depending on which PVT afferents were modulated.

Recent anatomical evidence indicates that the assumption that subpopulations of projection-specific neurons in the PVT control a specific response at the exclusion of others may be too simplistic. For instance, complete tracing of axons from a few PVT neurons using the Sindbis viral vector shows that the neurons mapped have axons that bifurcate extensively to innervate multiple regions of the forebrain (Unzai et al. 2015). Combination of injections of uniquely tagged cholera toxin B (CTB) in the NAcSh, BSTDL and CeL in the same animal demonstrates that many of the same neurons in the PVT provide collateral innervation to all three of these regions (Dong et al. 2017). These findings are consistent with the understanding that some populations of neurons have axons that provide branches called collaterals that innervate anatomically distant regions of the brain (Rockland 2018). Collateral innervation of multiple targets by PVT neurons suggests that this thalamic nucleus may have more complex effects on behavior than just having a projection-specific influence on a singular type of response. A notable finding of this latter tracing study was that neurons that project to different regions of the NAcSh arise from intermixed populations located in all regions of the PVT with those projecting to the dmNAcSh originating predominately from the anterior half of the PVT (aPVT) while those projecting to the vmNAcSh coming most frequently from the posterior half of the PVT (pPVT). Another notable finding from our previous study was that most PVT neurons were found to project to either the dmNAcSh or vmNAcSh (Dong et al. 2017). The fact that the neurons that projected to vmNAcSh, BSTDL, and CeL were primarily located in the pPVT pointed to the possibility that a group of neurons in the pPVT could form a highly collateralized projection system involved in aversive behavior (Dong et al. 2017). The present study involves a series of experiments to test the general premise of this hypothesis and to further characterize the extent that neurons in the PVT provide collateral innervation to multiple forebrain regions. First, experiments involving triple injections of CTB were done to test the hypothesis that neurons in the PVT that project to the vmNAcSh provide collaterals to the BSTDL and CeL. Second, an intersectional anterograde viral tracing approach was used to provide a better picture of the extent that PVT neurons that project to a given area give off collaterals to other areas of the forebrain. Third, experiments combining retrograde tracing with immunolabeling for the protein product of the immediate early gene cFos was used to determine if PVT neurons that project to the CeL or the dmNAcSh are differentially activated by aversive conditions. The overall purpose of the study was to generate a richer understanding of the organization of PVT neurons and their projection patterns in addition to determining if there is rudimentary evidence that an aversive experience preferentially activate subpopulations of PVT neurons.

## Methods

### Animals

A total of 64 male Sprague–Dawley rats (University of Manitoba vivarium) were used to generate the figures and data presented in this study. Rats weighing 250 ± 10 g were housed on a 12:12 h light–dark cycle with food and water freely available. Rats used for the behavioral experiments were handled for 2 min on alternate days during a 7-day adaptation period and all the behavioral procedures were done in the light cycle of the day (09:00–17:00). All experiments were carried out according to guidelines of the Canadian Council on Animal Care and approved by Research Ethics Review Board of the University of Manitoba.

### Retrograde tracing experiments

**Fig. 1:**
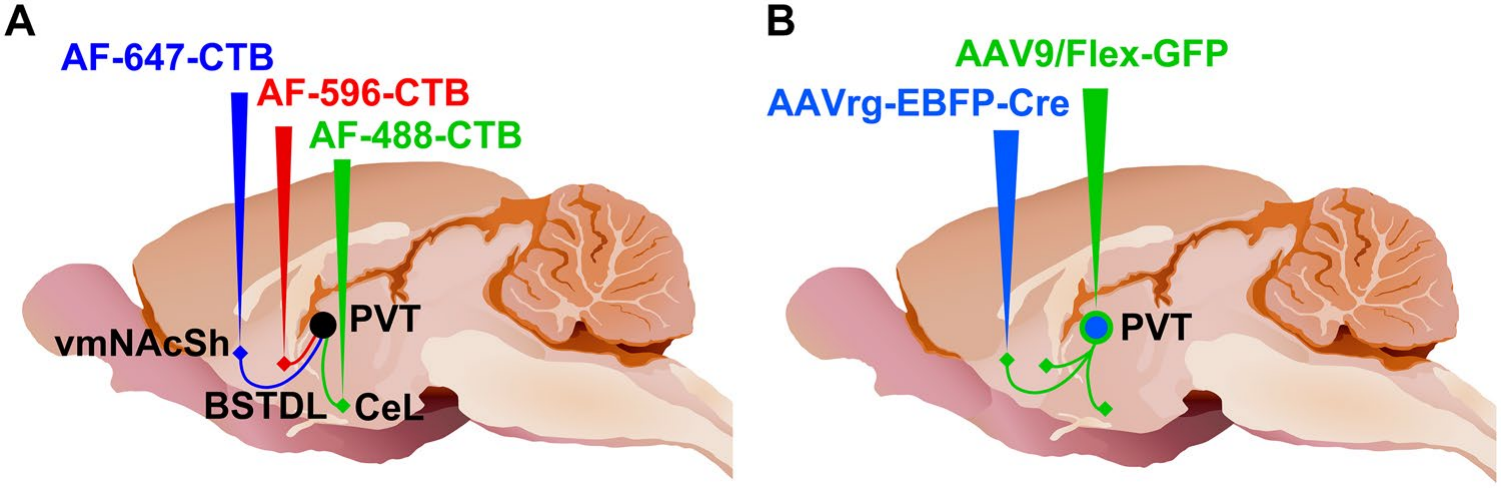
Diagram representing the location of injections for the retrograde triple-injection experiments **a** and the intersectional anterograde tracing experiments **b**. Injections of the CTB labeled with different fluorophores were placed in the vmNAcSh, BSTDL and CeL of the same animal (**a**). Injections of AAVrg-Syn1-EBFP-Cre were placed in one of the areas innervated by the PVT whereas injections of AAV9/Flex-GFP were placed in both the aPVT and pPVT (**b**). The sagittal brain image was created by Gill Brown, King’s College, London

The rats were anesthetized with 2–3% isoflurane and given meloxicam (2 mg/kg, s.c.) for post-surgery pain management. The animals were placed in a Stoelting stereotaxic frame and a hand drill was used to expose the brain surface above the target sites. Pressure injections of retrograde tracers were done using glass pipettes with outer diameter of approximately 37–40 µm. As illustrated in Fig. 1a, injections of the retrograde tracer CTB conjugated to Alexa Fluor 488 (AF-488-CTB, C22841, Invitrogen, Carlsbad, CA, USA), Alexa Fluor-594 (AF-594-CTB; C22842, Invitrogen) and Alexa Fluor 647 (AF-647-CTB; C34778, Invitrogen) were done in the vmNAcSh, BSTDL, CeL of the same animal (*n* = 4) using coordinates derived from a stereotaxic atlas of the rat brain (Paxinos and Watson 2009): for the vmNAcSh, 1.4 mm anterior, 1.3 mm lateral, 8.2 mm ventral; for the BSTDL, 0 mm anterior, 1.6–1.7 mm lateral, 6.7 mm ventral; and for the CeL, 2.4 mm posterior, 4.3 mm lateral, 8.2 mm ventral (all coordinates are relative to bregma bone surface). Solid CTB was dissolved in 0.06 M neutral phosphate buffer (PB) at 0.5% concentration and injected at a volume of 50 nl over 10–15 min using a picospritzer on the same side of the brain. The scalp incisions were sutured and rats were returned to their home cages for recovery for 8 to 9 days to allow sufficient time for retrograde transport and accumulation of CTB in PVT neurons.

Animals were deeply anesthetized with 10% chloral hydrate (600 mg/kg, i.p.) and perfused transcardially with 150 ml heparinized saline followed by 400–500 ml ice-cold 4% paraformaldehyde in 0.1 M PB (pH 7.4). The brains were removed and post-fixed in the same fixative overnight and cryoprotected in PBS with 20% sucrose and 10% glycerin over 2 days at 4 °C. Coronal sections of the brain containing the injection sites and the PVT were taken with a cryostat (UltroPro 5000) at 50 µm, mounted at every 200 µm on slides and coverslipped with fluorescent protectant mounting media Fluoromount-G (SouthernBiotech, Birmingham, AL, USA) for subsequent examination.

### Intersectional anterograde tracing experiments

Animals were prepared as above and placed in the stereotaxic frame and injected using procedures similar to the retrograde tracing experiments. As shown in Fig. 1b, subgroups of rats received injections in a region of interest (ROI) of an adeno-associated virus (AAV) that transduces neurons in a retrograde direction to express Cre-recombinase (AAVrg-Cre) and injections of a Cre-dependent AAV that transduces neurons to express GFP in an anterograde direction (AAV9/Flex-GFP) in the PVT to label the fibers associated with the PVT neurons that project to the ROI (defined as the primary projection) and other forebrain regions (defined as collateral projections). Injections of a 300 nl solution of AAVrg-Syn1-EBFP-Cre (7.6 × 10^12^ GC/ml; #51507-AAVrg, Addgene, Cambridge, MA, USA) were done in the following areas: the vmNAcSh, 1.4 mm anterior, 1.3 mm lateral, 8.2 mm ventral; the dmNAcSh, 1.5 mm anterior, 0.9 mm lateral, 6.8 mm ventral; the BSTDL, 0 mm anterior, 1.6 mm lateral, 6.7 mm ventral; the CeL, 2.1 mm posterior, 4.3 mm lateral, 8.2 mm ventral; the dorsomedial nucleus of the hypothalamus, 3.2 mm posterior, 2.0 mm lateral, 8.9 mm ventral, angled at 10°; and the infralimbic cortex, 3.0 mm anterior, 1.4 mm lateral, 5.0 mm ventral, angled at 10°. All animals received an injection of 500 nl of an AAV9/Flex-GFP solution (5.21 × 10^13^ GC/ml, Salk Institute Viral Vector Core, La Jolla, CA, USA) in both the aPVT (1.8 mm posterior, 1.0 mm lateral, 5.9 mm ventral, angle at 10°) and the pPVT (3.1 mm posterior, 1.0 mm lateral, 5.8 mm ventral, angle at 10°). All coordinates are relative to bregma bone surface. The pipette was kept in place for 10 min after the injections before it was slowly withdrawn. The scalp incisions were sutured and rats were returned to their home cages and kept for a 4-week period to maximize transport and transduction of Cre-recombinase and the fluorescent proteins.

Animals were perfused as before and coronal sections of the forebrain were taken at 50 µm. Every sixth section was incubated in a blocking solution of 5% donkey serum, 0.3% of Triton X-100 and 0.1% of sodium azide for one hour. Sections were then transferred to a primary antibody cocktail containing rabbit anti-GFP antibody (1:1000; A11122, Invitrogen) and mouse anti-Cre antibody (1:1000; MAB3120, Millipore, Temecula, CA, USA) in the blocking solution overnight. After several rinses, sections were transferred to a secondary antibody cocktail solution containing Alexa-Fluor 488 donkey anti-rabbit antibody (1:1000; A21206, Invitrogen) and Cy3 donkey anti-mouse antibody (1:500; 715-165-151, Jackson Immunoresearch, West Grove, PA, USA) for 2 h. The sections were rinsed with PBS and mounted on slides and then coverslips were applied with Fluromount-G.

### cFos expression to aversive conditions experiments

#### Open field exposure

The open field test was done in a Plexiglas box (80 × 80 × 40 cm). Rats were randomly assigned to one of three conditions: open field exposure at 1 or 100 lux illumination in the center (*n* = 6 for each group) or home cage control (*n* = 4). Rats were placed in a corner of the open field and allowed to explore for 10 min. Their behavior in the open field was videotaped by a camera mounted on the ceiling. The time spent in the center area (35 × 35 cm) and the total distance traveled were analyzed by a software Ethovision (Noldus, Wageningen, Netherland). The control group involved of rats being moved from the colony room to the test room in the same way as the animals used for the open field test but were kept in their covered home cages for 10 min and were not directly physically handled by the experimenters. Rats were anesthetized 90 min after being returned to their home cages in the colony room and perfused with fixative as described above.

#### Footshock exposure and contextual fear memory retrieval

Animals were prepared for stereotaxic surgery as above and a combination of AF-594-CTB and AF-488-CTB was injected in the dmNAcSh (1.5 mm anterior, 0.9 mm lateral, 6.8 mm ventral) and CeL (2.4 mm posterior, 4.3 mm lateral, 8.2 mm ventral) of the same animal on the same side of the brain using the same procedures as described for the triple-retrograde tracing experiments (*n* = 24). One week after the stereotaxic injections, groups of injected rats were placed in a shock chamber (MED Associates, St. Albans, Vermont, USA) for 5 min and received footshocks (5 × 2 s of 1.5 mA after 2 min of acclimation time, *n* = 6) or received no shocks (*n* = 6) and then placed in their home cage for 90 min before being anesthetized and perfused with fixative as described above. Another group of shocked (*n* = 6) and nonshocked rats (*n* = 6) that had also received combination of injections of CTB in the dmNAcSh and CeL were re-exposed to the shock chamber the next day and returned to their home cage for 90 min before being anesthetized and perfused with fixative. The amount of freezing displayed in the shock chamber was quantified to determine if shock context re-exposure induced conditioned contextual fear expression.

#### Immunoreactions for cFos

Brain sections containing the PVT region were cut on a cryostat and pre-incubated in the blocking solution for 1 h and then incubated in primary rabbit anti-cFos antibody (1:2000; ABE457, Millipore) overnight. After 3 rinses in PBS, sections were transferred into a secondary donkey anti-rabbit conjugated to Alexa-Fluor 647 (1:1000; A31573, Invitrogen) antiserum for 2 h. After three more rinsing steps, sections were mounted and coverslipped as described above.

### Image and quantitative analysis

Brain sections were examined and photographed using a Zeiss Axio Observer Z1 microscope equipped with Axiocam 503 mono camera. The images showing the distribution of neurons in the PVT or fiber projections to areas of the brain innervated by the PVT are stacks of images compiled using Zen Blue Software (Zeiss). The contrast of the composite images was adjusted in Adobe Photoshop to produce the final images shown in the publication.

#### Retrograde tracing experiments

Images were taken under 20 × magnification objective lens, and numbers of X and Y stacks were set to cover the ROI. The exposure time was adjusted for each individual channel to optimize the images captured and was set at a consistent level for all different cases. The images were processed with Zen Blue Software using the “Stitch” application for fusing the X and Y stacks. Retrogradely labeled CTB positive neurons in the PVT were quantified and mapped under the same gamma setting for each color channel. Neurons labeled with different CTB-fluorophore were manually marked and counted using the software under appropriate filter as single-labeled neurons. Similarly, the double- and triple-labeled PVT neurons were manually marked by first identifying them under dual- and triple- filters and verifying the marked designation by examining the neuron under individual filters when the merged signal was ambiguous. The number of CTB-labeled neurons in the PVT from injections in the ROI was quantified from the digital images captured of the midline thalamus. The presence of a specific color tag was marked manually on images of coronal sections of the midline thalamus of every 360 µm starting at the level of the aPVT (1.08 mm posterior to bregma) to the pPVT (3.60 mm posterior to bregma) according to the rat brain atlas (Paxinos and Watson 2009). For experiments involving quantification of cFos expression, the number of neurons labeled with cFos immunofluorescence and various CTB fluorophores is reported as the number of neurons from the aPVT (1.08 to 2.40 posterior to bregma) and the pPVT (2.52 to 3.72 mm posterior to bregma).

The images with the marked single-, double- and triple-labeled neurons were imported to Adobe Illustrator CS4 and merged with an image file of the appropriate anatomical level of the thalamus from the digital atlas of the rat brain (Paxinos and Watson 2009). The shape and orientation of neurons and fibers labeled with CTB was used to identify the boundaries of the PVT from adjacent nuclei. The boundaries of the thalamic nuclei from the atlas were adjusted slightly to correspond with the microscopic image. The merged image was exported to Adobe Photoshop and the locations of the labeled neurons in the thalamus were generated by manually placing the appropriate color labeled dot at the location of the labeled neuron in the merged image. The final image files of individual stereotaxic levels were used to compile the figures showing the retrograde labeling in the PVT.

#### Anterograde tracing experiments

Representative regions of the forebrain with high GFP fiber labeling and known to be innervated by the PVT were taken under 10 × magnification. Digital images were imported to Adobe Photoshop to optimize light and contrast levels for generation of figures showing the fiber staining and for drawing of the location of the AAVrg-Cre injections. The number of neurons in the PVT expressing GFP in the anterograde tracing experiments was quantified using the same methods as described for the retrograde tracing experiments. The density of fibers in the different areas innervated by the PVT was quantified from images taken using a Zeiss LSM 880 confocal microscope. All confocal images were taken using the same setting under 20 × magnification with Airyscan with 9 Z-stack set at 2 µm intervals. Figure 13 illustrates the areas sampled that were selected based on the presence of PVT fibers as observed in the present study and a previous anterograde tracing experiments by our group (Li and Kirouac 2008). It should be understood that the areas sampled using the confocal microscope were not unbiased in that the areas were selected because of the presence of a high (e.g., areas of the extended amygdala) or moderate density of labeled fibers (e.g., areas outside the extended amygdala) and taken where there was a homogenous distribution of GFP fibers. The captured images were then processed using the Zen applications *Airyscan* and *Maximum Intensity of Projection* for analysis. Fiber density was quantified from the confocal images using an open source image processing package based on ImageJ (Fiji) according to previously described methods where labeled axons are detected and converted to pixel per area imaged (Grider et al. 2006). Briefly, the plug-in application *Feature J* was used to detect linear structures in the confocal images using Hessian-based edge detection and converted into 8-bit files with the threshold set at the same value to calculate the pixel density of each images. This resulted in binary images of GFP axons with the background fluorescence eliminated. The number of pixels corresponding to positive GFP labeling was summed for each ROI and the proportion of pixels covering the area of a confocal image analyzed is reported as the percent innervation density.

### Statistical analyses

The number of neurons, fiber density, and behavioral measures were analyzed using one-way or two-way ANOVA followed by post-hoc Tukey tests to identify group differences using the software OriginPro 8 (OriginLab Corporation, Northampton, MA, USA). The percentage of the total number of neurons activated in the aPVT and pPVT was determined by dividing the number of cFos nuclei in the PVT by the number of neurons in the aPVT and pPVT according to counts of NeuN positive neurons in PVT from our previous study where the average numbers of NeuN-stained neurons was found to be 7412 for the aPVT and 5854 for the pPVT (Dong et al. 2017). An adjusted value of *p* < 0.05 was considered to be significant and the data are presented as mean ± SEM.

## Results

### Retrograde tracing experiments

**Fig. 2:**
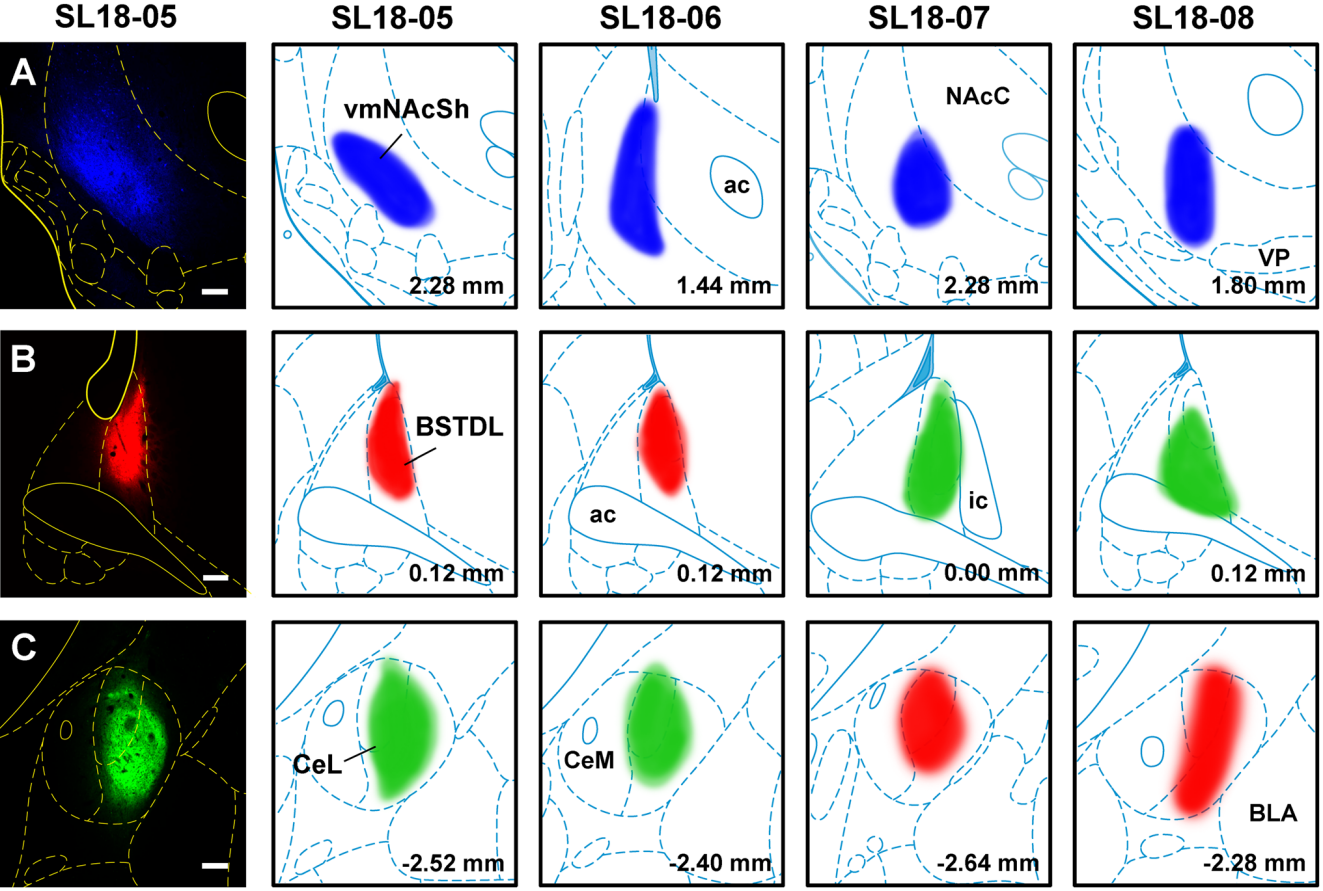
Diagram showing the location of CTB injections in the vmNAcSh, BSTDL, and CeL for the cases used for the triple-injection retrograde tracing experiments. Rows show that the injections were confined to the vmNAcSh (**a**), BSTDL (**b**) and the CeL (**c**) in 4 animals (columns) at the stereotaxic level known to receive dense innervation from the PVT (Li and Kirouac 2008). See list for abbreviations. Numbers at the bottom represent distance from the bregma. Scale bars: 200 µm

**Fig. 3:**
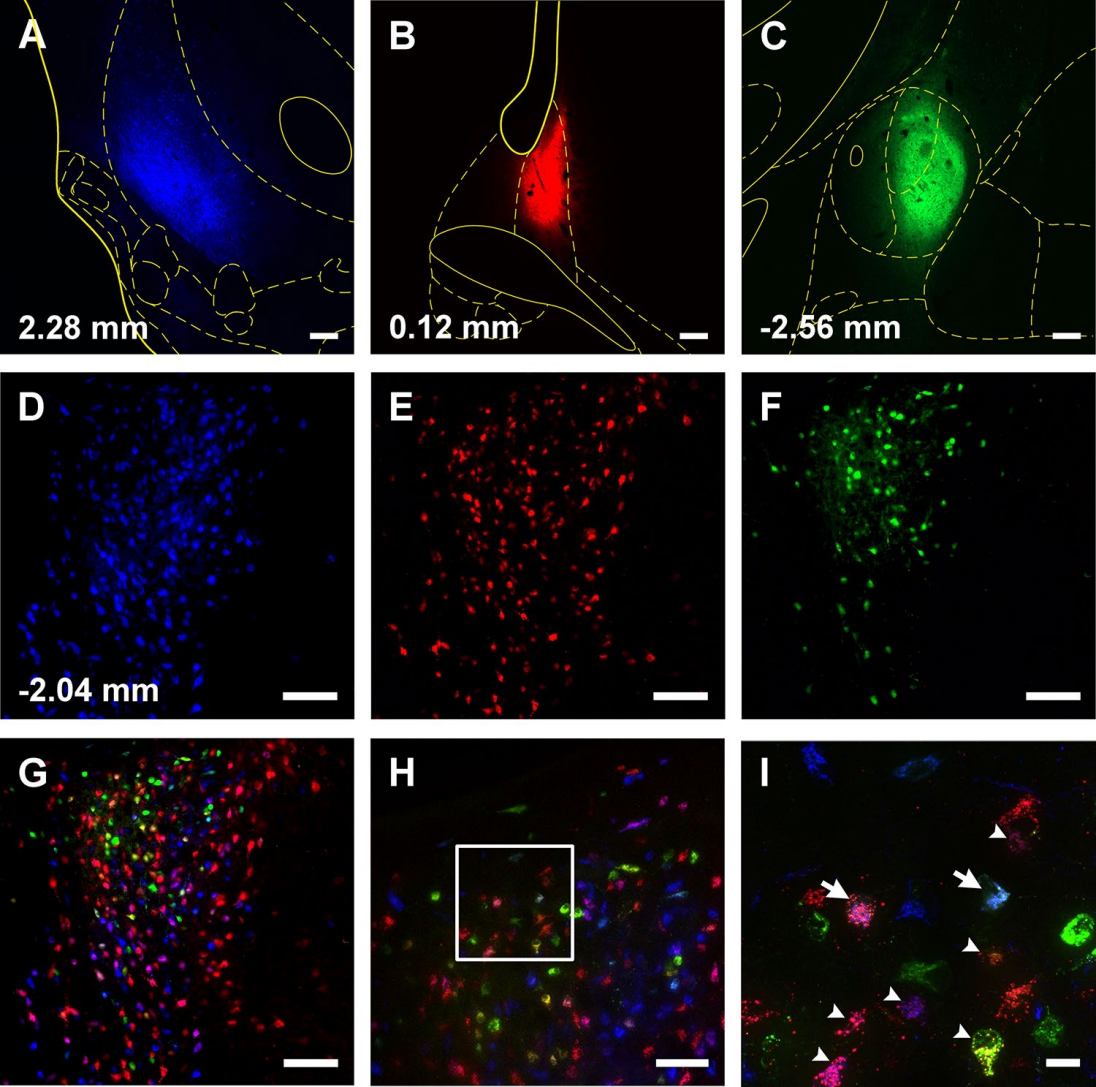
Representative example of one case with injections of CTB in the vmNAcSh (**a**), BSTDL (**b**) and CeL (**c**) with the corresponding retrograde labeling in one stereotaxic level of the aPVT for each different fluorophore (**d**–**f**). Higher magnifications (**h**) of the same area of the merged image (**g**) shows numerous double-, and triple-labeled neurons in the aPVT. The arrowheads in the inset (**i**) indicate examples of double-labeled neurons while the arrows indicate triple-labeled neurons. Numbers at the bottom represent distance from the bregma. Scale bars: **a**–**c**, 200 µm; **d**–**g**, 200 µm; **h**, 100 µm; **i**, 10 µm

**Fig. 4:**
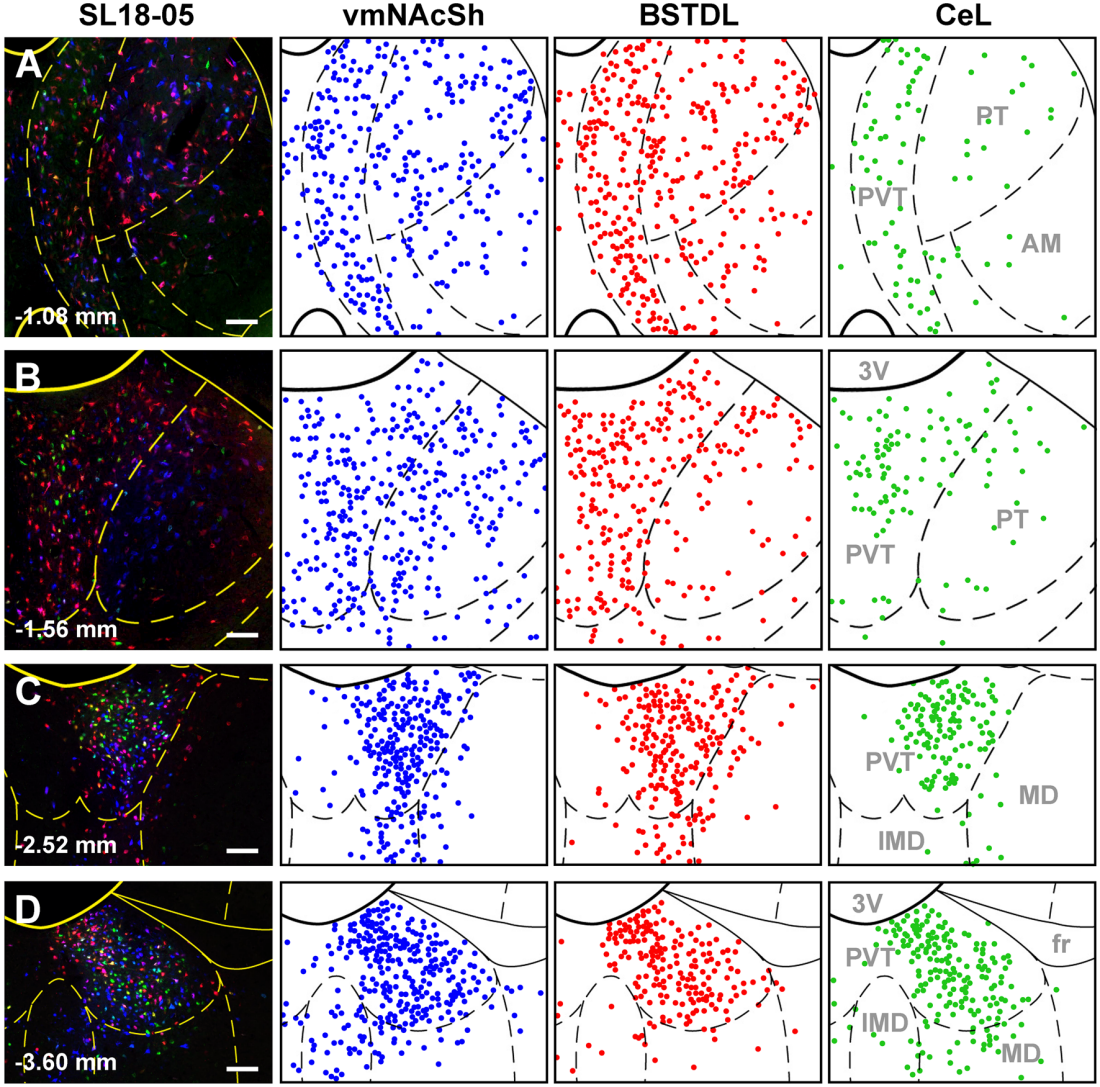
Distribution of single-labeled neurons in the PVT from injections of CTB in the vmNAcSh, BSTDL, and CeL in one representative case (SL18-05). Rows show the labeling observed in the anterior most aspect of the PVT (**a**), middle regions (**b**, **c**) and posterior most aspect of the PVT (**d**) arranged according to the site of the injections in each target areas as shown in the columns (color coded according to the fluorophore attached to the CTB). See list for abbreviations. Numbers at the bottom represent distance from the bregma. Scale bars: 200 µm

**Fig. 5:**
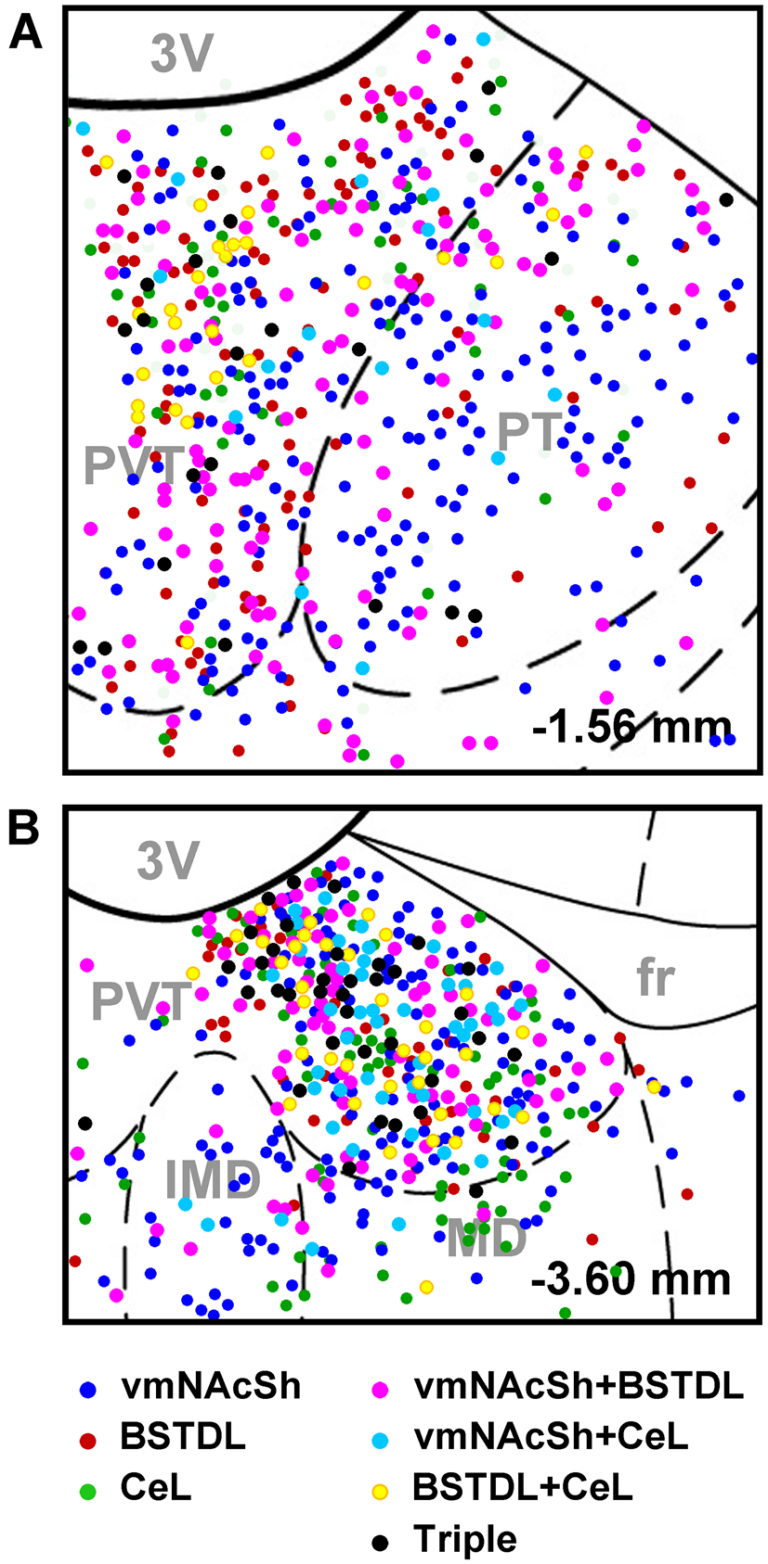
The distributions of single-, double- and triple-labeled neurons in the PVT from injections of CTB in the vmNAcSh, BSTDL, and CeL in case SL18-05 are shown for representative stereotaxic levels of the aPVT (**a**) and pPVT (**b**). See list for abbreviations. Numbers at the bottom represent the distance from the bregma

**Fig. 6:**
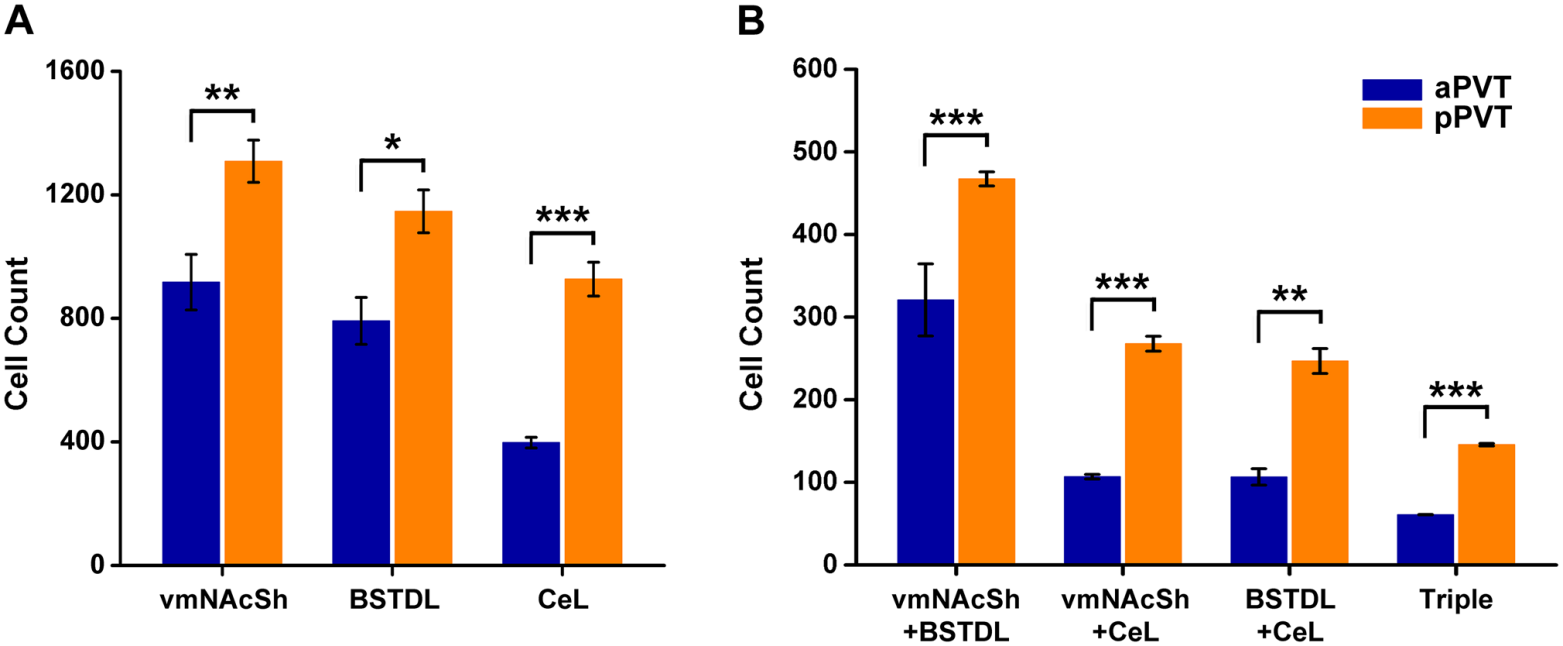
Quantitative analysis of the number of neurons in the aPVT and pPVT from injections of CTB in the vmNAcSh, BSTDL, and CeL. **a** Number of single-labeled neurons from injections in the three areas studied. **b** Number of double-labeled neurons (various combinations) and triple-labeled neurons in the aPVT and pPVT. *p < 0.05, **p < 0.01, ***p < 0.001

Four cases with injections of CTB clearly within the vmNAcSh, BSTDL and CeL were used for generating the figures and for data analysis (Fig. 2). The criteria for the selection of a case for analysis was that the diffusion of the CTB was of a similar extent for the vmNAcSh, BSTDL and CeL in addition to an absence of discernable CTB diffusion in anatomical structures adjacent to the targeted area. Figure 3 shows the size and location of CTB injections in the vmNAcSh, BSTDL and CeL of one representative case (SL18-05) along with examples of the labeled neurons in the aPVT. Single-, double- and triple-labeled neurons are intermixed throughout the aPVT and do not form discernable clusters. Figure 4 displays the location of single-labeled neurons in the anterior to posterior extent of the PVT and the adjacent nuclei in the dorsal midline thalamus from the representative case. Neurons labeled from injections in the vmNAcSh were located throughout the PVT with the density of labeled neurons increasing at more posterior levels. Similar patterns were observed for injections in the BSTDL and CeL albeit with a more noticeable anterior–posterior gradient culminating with the highest density in the posterior most level of the PVT (i.e., traditionally called the pPVT). Injections of CTB in the vmNAcSh also resulted in a number of labeled neurons scattered in the paratenial nucleus as previously reported (Dong et al. 2017; Li and Kirouac 2008). Figure 5 illustrates the location of single-, double- and triple-labeled neurons for one representative level of the aPVT and pPVT produced by injections of CTB in the vmNAcSh, BSTDL and CeL from the representative case SL18-05. Double- or triple-labeled neurons were found scattered and intermixed with single-labeled neurons throughout the PVT and without forming clusters of distinct populations except for the apparent high density of multi-labeled neurons in the pPVT. The total number of neurons labeled from these injections was quantified and analyzed to detect differences between the aPVT and pPVT (Fig. 6). There was a significant difference in the number of single-labeled neurons originating from the aPVT and pPVT (Fig. 6a, *F*_(1,20)_ = 61.40, *p* < 0.001) with higher numbers observed in the pPVT for injections in vmNAcSh, BSTDL and CeL as indicated by Tukey post-hoc analysis tests. Similar findings were found when considering the number of double-labeled neurons in the aPVT and pPVT (Fig. 6b, *F*_(1,20)_ = 91.54, *p* < 0.001) with the numbers of retrograde-labeled neurons being greater in the pPVT for the various combinations of the injections (significance levels for the post-hoc tests are reported in the figure legend). The number of triple-labeled neurons was also found to be greater in the pPVT (Fig. 6b, *F*_(1,6)_ = 3105.97, *p* < 0.001). In summary, there were more single-, double- and triple-labeled neurons in the pPVT than were in the aPVT from injections of CTB in the vmNAcSh, BSTDL, and CeL. These findings are consistent with our previous study using various combinations of double CTB injections in these three regions (Dong et al. 2017).

### Intersectional anterograde tracing experiments

The main purpose of the anterograde experiments was to determine if a ROI (dmNAcSh, vmNAcSh, BSTDL and CeL) receives fiber projections from unique populations of PVT neurons that project primarily to the ROI (evidence for projection-specific neurons) or from neurons that project to other areas of interest (evidence for collateral innervation). To facilitate the presentation of the results and the discussion, we make reference to neurons that provide a primary projection to ROI as those neurons transduced with the injection of the AAVrg-Cre in the ROI whereas neurons that provide a collateral projection to the ROI as the neurons that were transduced by injections of the AAVrg-Cre in other areas targeted in different rats. However, it should be clear that it is likely that most PVT neurons have axons that bifurcate to provide innervation to different regions of the forebrain so that all the projection fibers studied here are collateral projection (Unzai et al. 2015).

**Fig. 7:**
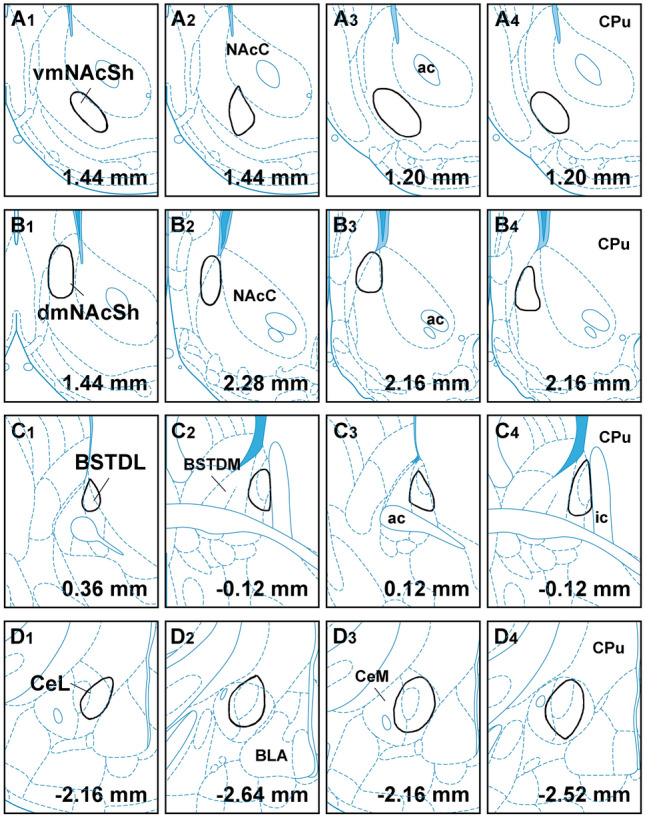
Location of AAV-Cre injections in the vmNAcSh (**a**), dmNAcSh (**b**), BSTDL (**c**) and CeL (**d**) for the intersectional anterograde tracing experiments. The approximate locations of the injection sites were identified by the presence of Cre in neurons at the site of the injections. See list for abbreviations. Numbers at the bottom represent distance from the bregma

**Fig. 8:**
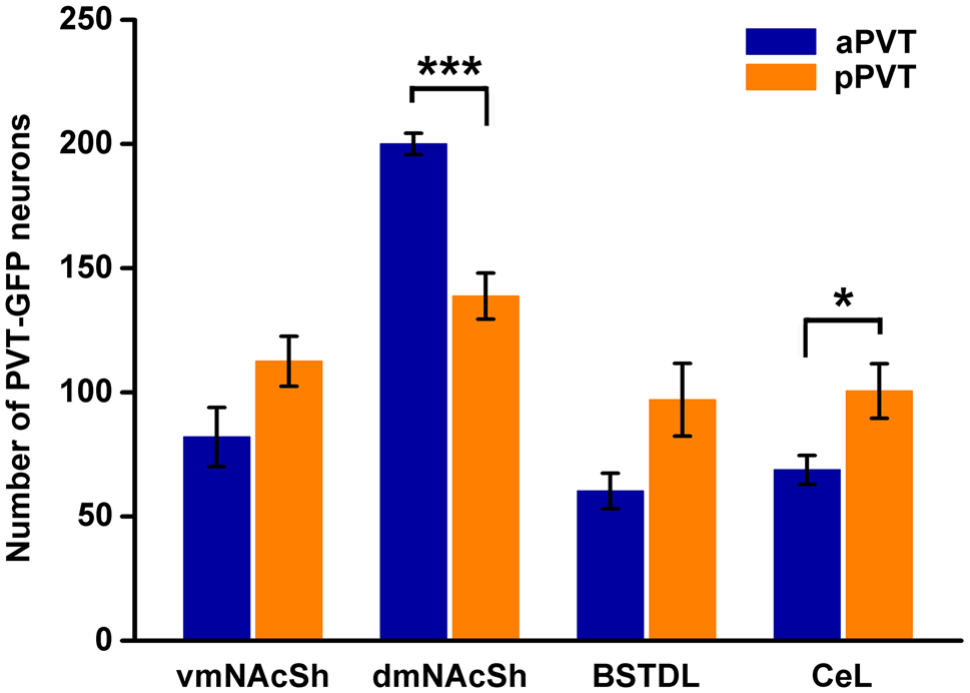
Number of neurons in the aPVT and pPVT transduced in the intersectional anterograde tracing experiments. *n* = 4 for each group, *p < 0.05, ***p < 0.001

Figure 7 illustrates the location of the AAVrg-Cre injections in the 16 cases used for the analysis of the innervation density provided by the primary and collateral projections. Figure 8 shows the number of neurons in the aPVT and pPVT transduced by the intersectional approach. There was a significantly greater number of neurons transduced in the aPVT than in the pPVT in the cases that had received injections of AAVrg-Cre in the dmNAcSh (*F*_(1,6)_ = 35.88, *p* < 0.001) whereas more neurons were transduced in the pPVT than that of the aPVT in the cases with injections of AAVrg-Cre in the CeL (*F*_(1,6)_ = 6.51, *p* = 0.043). The pattern of labeling observed in the aPVT and pPVT using the intersectional approach is consistent with what is known about the location of PVT neurons that project to the NAcSh, BSTDL and CeL (Dong et al. 2017; Li and Kirouac 2008) validating the intersectional anterograde approach.

**Fig. 9:**
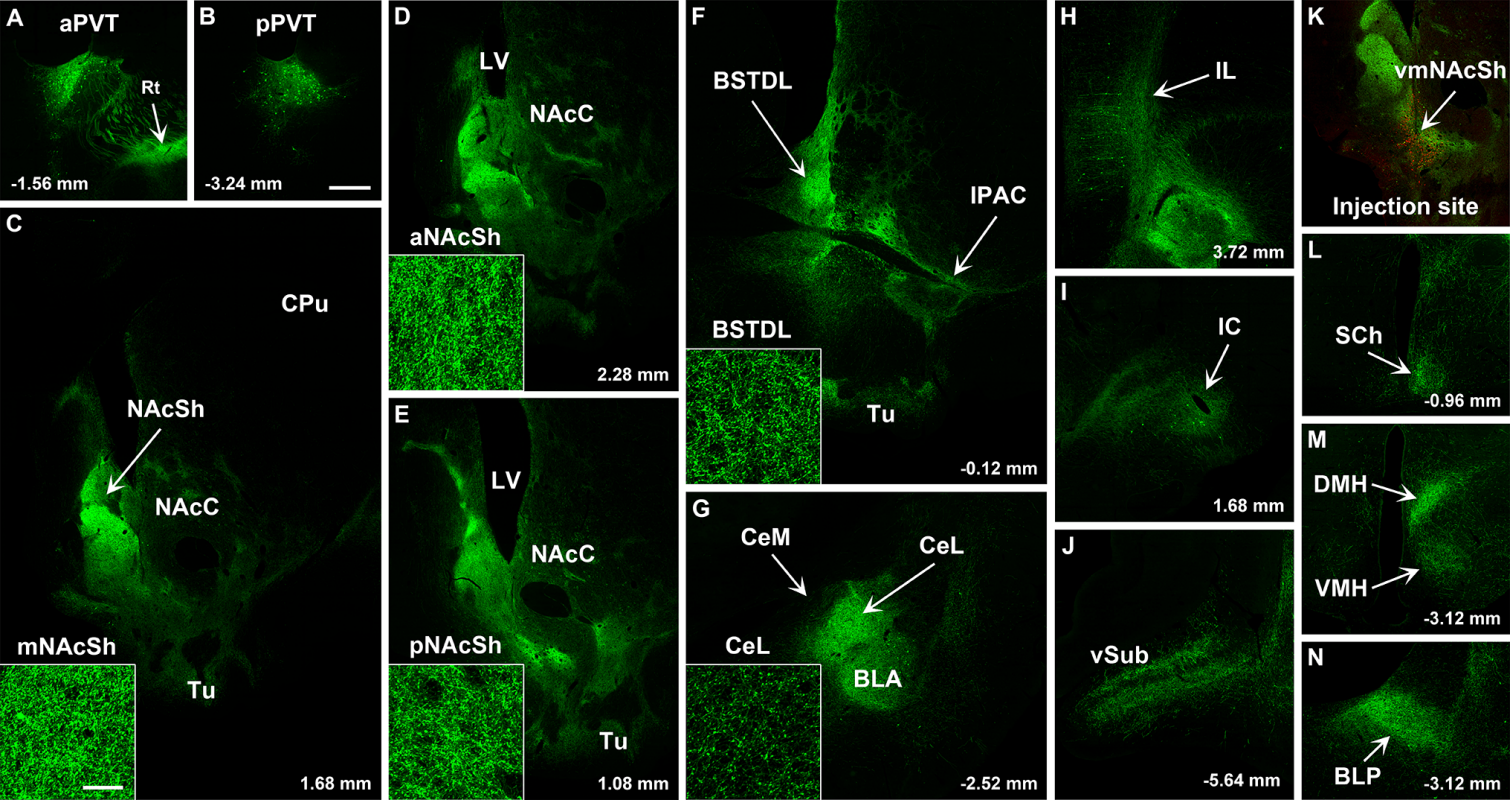
Images of regions of the forebrain receiving significant innervation from PVT transduced by injections of AAVrg-Cre in the vmNASh (**k**, red immunofluorescence for Cre) and AAV-GFP in the PVT. Images of the aPVT (**a**) and pPVT (**b**) showing GFP-transduced neurons and resulting fiber labeling in the middle (**c**), anterior (**d**), posterior aspect (**e**) of the NAcSh, BSTDL (**f**), CeL (**g**), infralimbic cortex (**h**), insular cortex (**i**), ventral subiculum (**j**), suprachiasmatic nucleus (**l**), medial hypothalamus (**m**), and posterior nucleus of the amygdala (**n**). See list for abbreviations. Scale bars: 500 µm for all panels and 10 µm for insets of the high magnification confocal images. Numbers at the bottom represent distance from the bregma

**Fig. 10:**
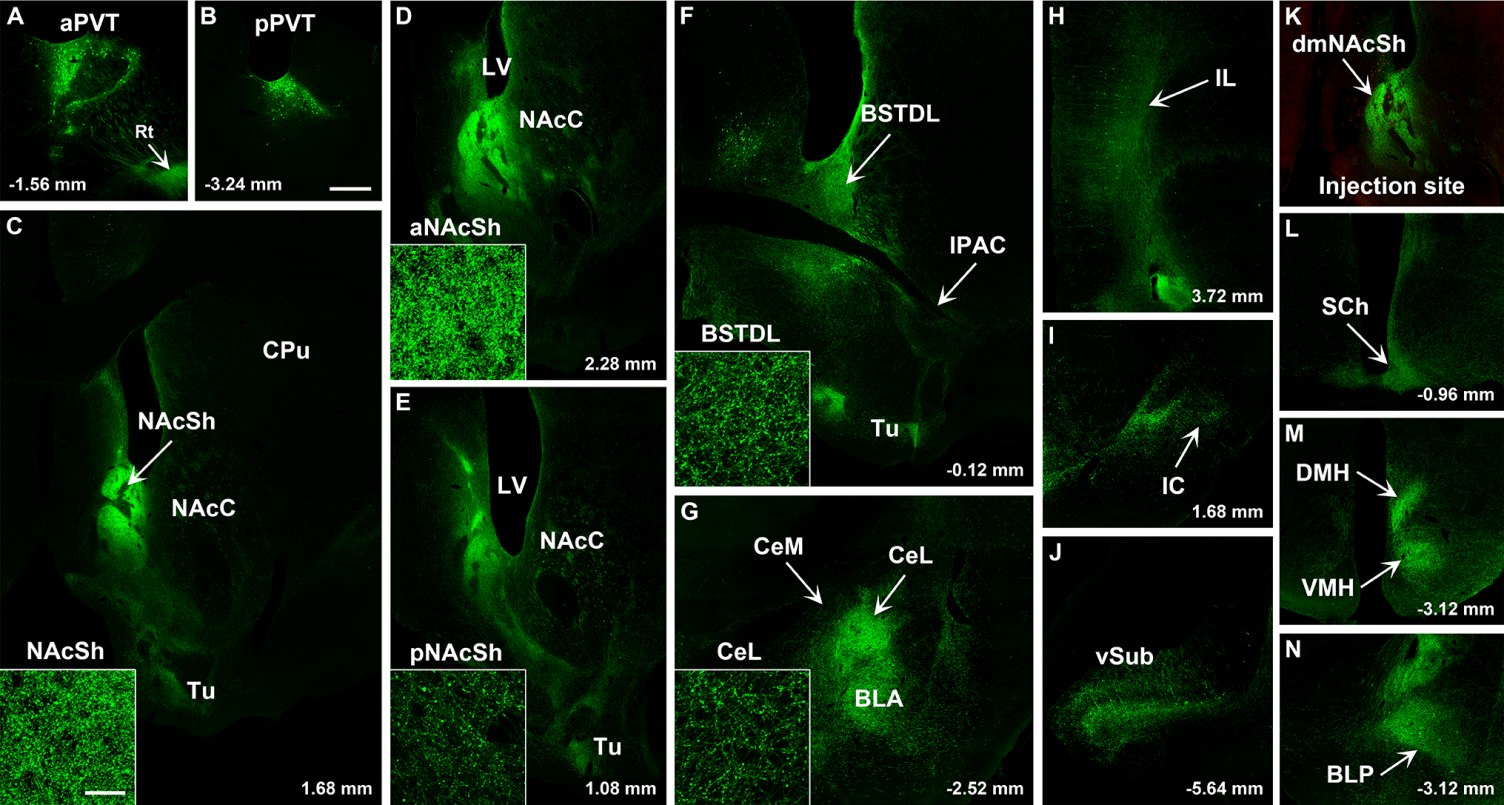
Images of regions of the forebrain receiving significant innervation from PVT transduced by injections of AAVrg-Cre in the dmNASh (**k**, red immunofluorescence for Cre) and AAV-GFP in the PVT. Images of the aPVT (**a**) and pPVT (**b**) showing GFP-transduced neurons and resulting fiber labeling in the middle (**c**), anterior (**d**), posterior aspect (**e**) of the NAcSh, BSTDL (**f**), CeL (**g**), infralimbic cortex (**h**), insular cortex (**i**), ventral subiculum (**j**), suprachiasmatic nucleus (**l**), medial hypothalamus (**m**), and posterior nucleus of the amygdala (**n**). See list for abbreviations. Scale bars: 500 µm for all panels and 10 µm for insets of the high magnification confocal images. Numbers at the bottom represent distance from the bregma

**Fig. 11:**
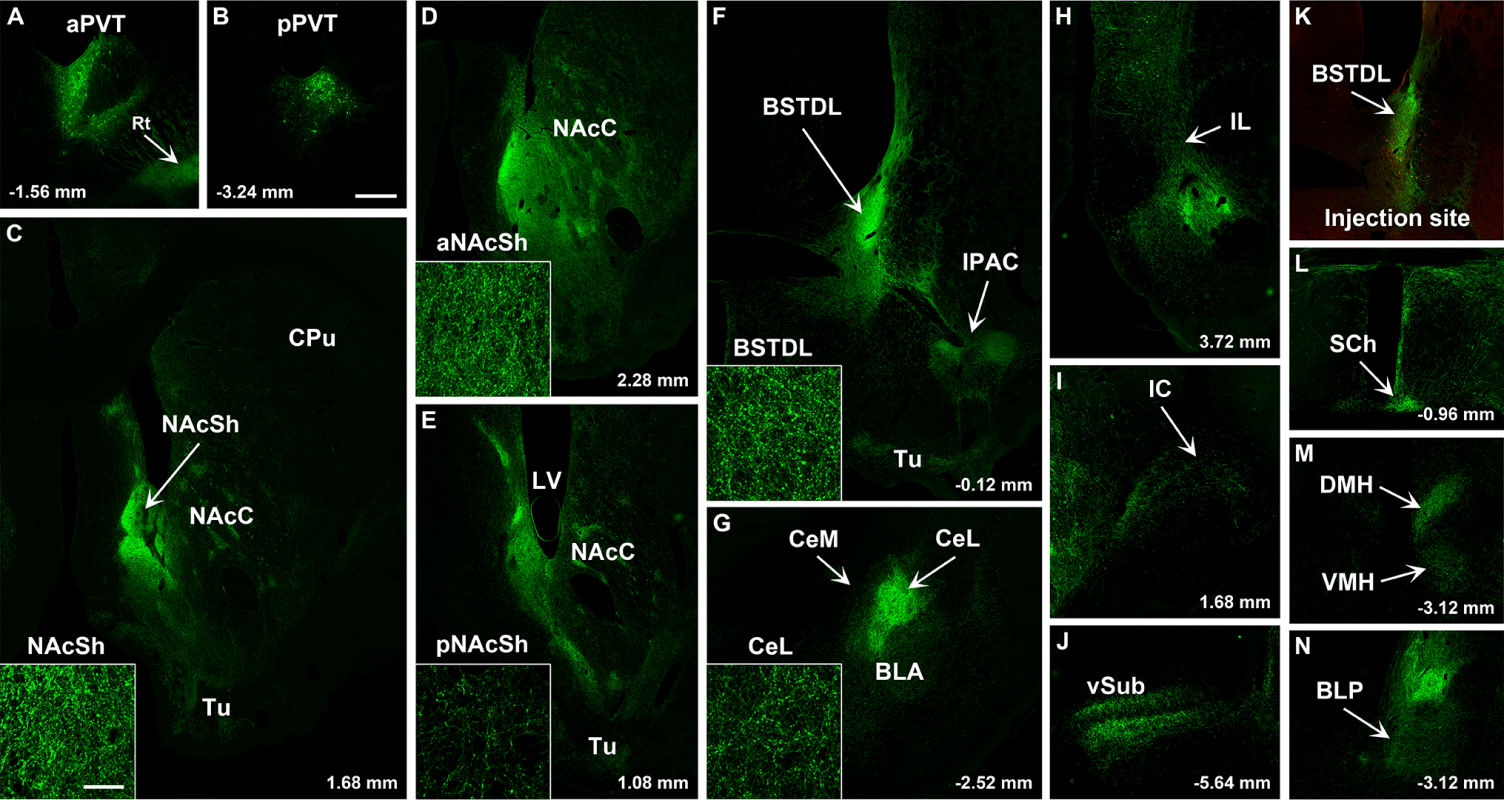
Images of regions of the forebrain receiving significant innervation from PVT transduced by injections of AAVrg-Cre in the BSTDL (**k,** red immunofluorescence for Cre) and AAV-GFP in the PVT. Images of the aPVT (**a**) and pPVT (**b**) showing GFP-transduced neurons and resulting fiber labeling in the middle aspect (**c**), anterior (**d**), posterior aspect (**e**) of the NAcSh, BSTDL (**f**), CeL (**g**), infralimbic cortex (**h**), insular cortex (**i**), ventral subiculum (**j**), suprachiasmatic nucleus (**l**), medial hypothalamus (**m**), and posterior nucleus of the amygdala (**n**). See list for abbreviations. Scale bars: 500 µm for all panels and 10 µm for insets of the high magnification confocal images. Numbers at the bottom represent distance from the bregma

**Fig. 12:**
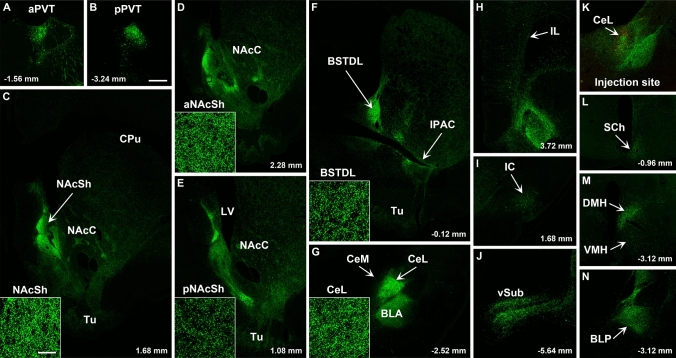
Images of regions of the forebrain receiving significant innervation from PVT transduced by injections of AAVrg-Cre in the CeL (**k,** red immunofluorescence for Cre) and AAV-GFP in the PVT. Images of the aPVT (**a**) and pPVT (**b**) showing GFP-transduced neurons and resulting fiber labeling in the middle aspect (**c**), anterior (**d**), posterior aspect (**e**) of the NAcSh, BSTDL (**f**), CeL (**g**), infralimbic cortex (**h**), insular cortex (**i**), ventral subiculum (**j**), suprachiasmatic nucleus (**l**), medial hypothalamus (**m**), and posterior nucleus of the amygdala (**n**). See list for abbreviations. Scale bars: 500 µm for all panels and 10 µm for insets of the high magnification confocal images. Numbers at the bottom represent distance from the bregma

Regardless of the area targeted by the AAVrg-Cre (dmNAcSh, vmNAcSh, BSTDL, or CeL), the pattern of GFP fiber labeling was remarkably similar except for some qualitative difference in fiber density in various forebrain regions innervated by the PVT (Figs. 9, 10, 11, 12). The pattern of innervation invariantly observed across the different cases can be summarized as follows. First, the intersectional approach transduced neurons in both the aPVT and pPVT (panels A and B in Figs. 9, 10, 11, 12). Second, a moderate to dense fiber density was observed in the dmNAcSh and vmNAcSh (panels C, D, E), BSTDL (panels F), or CeL (panels G) regardless of the location of the AAVrg-Cre. Dense fiber labeling was observed in the anterior (panels D), middle (panels C), and posterior (panels E) aspects of this region of the nucleus accumbens. Third, a light to moderately dense fiber labeling was seen in the same regions of the brain previously reported to receive fibers from the PVT using traditional anterograde tracers (Li and Kirouac 2008; Vertes and Hoover 2008). These included the core of the nucleus accumbens and olfactory tubercle (panels C, D, E), reticular nucleus of the thalamus (panel A), infralimbic-prelimbic cortex (panels H), insular cortex (panels I), interstitial nucleus of the posterior limb of the anterior commissure or IPAC (panels F), basolateral amygdala (panels G), ventral subiculum (panels J), suprachiasmatic nucleus of the hypothalamus (panels L), as well as the dorsomedial and ventromedial nuclei of the hypothalamus (panels M). Finally, relatively light labeling was also observed in many regions adjacent to those receiving the dense labeling including the caudate putamen (panels C, D, E); ventral and medial regions of the bed nucleus of the stria terminalis (panels F); and the basomedial and cortical nuclei of the amygdala (panel G).

**Fig. 13:**
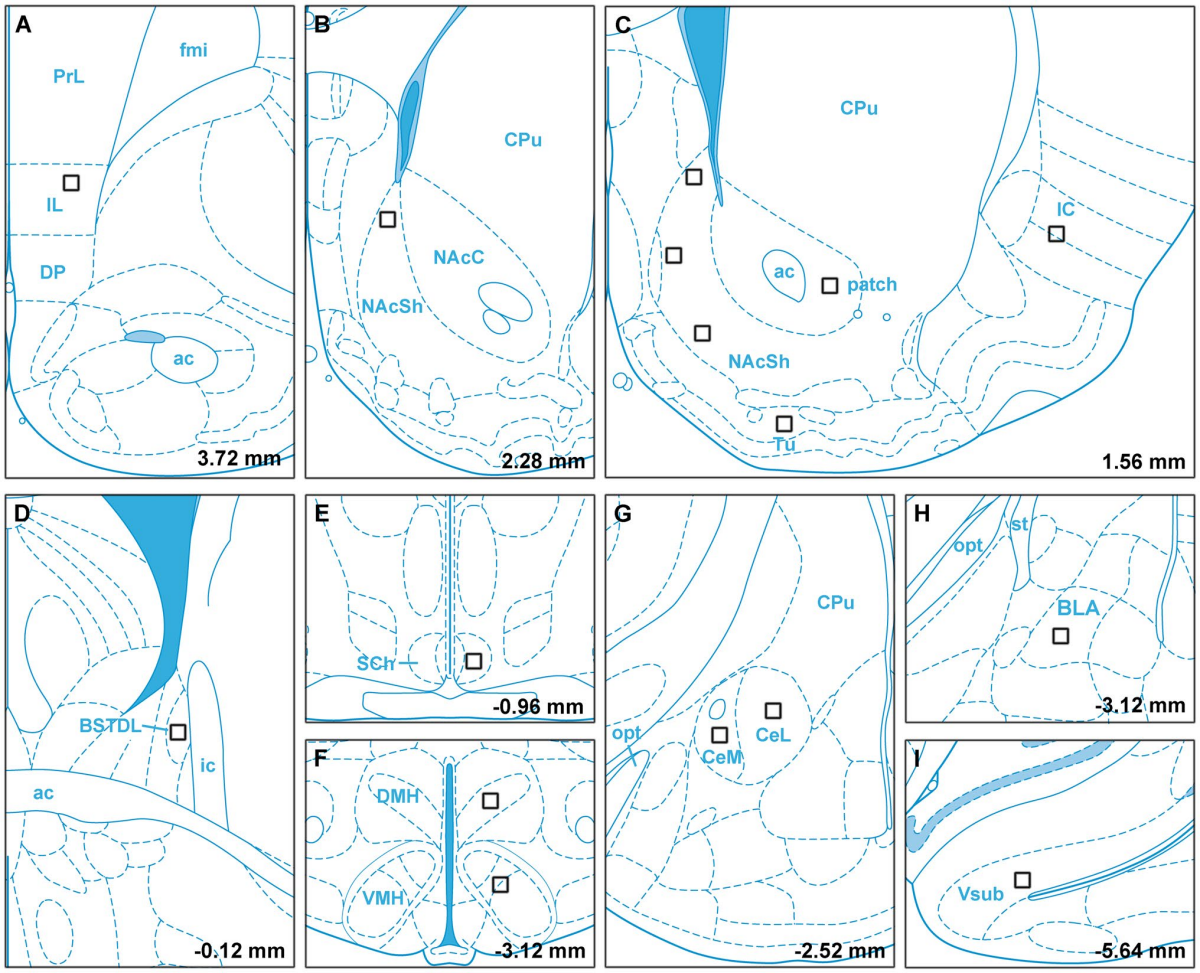
Diagram showing the location where confocal images were taken for quantitative analysis of fiber innervation density. See list for abbreviations. Numbers at the bottom represent distance from the bregma. Diagram adapted from a stereotaxic atlas (Paxinos and Watson 2009)

**Fig. 14:**
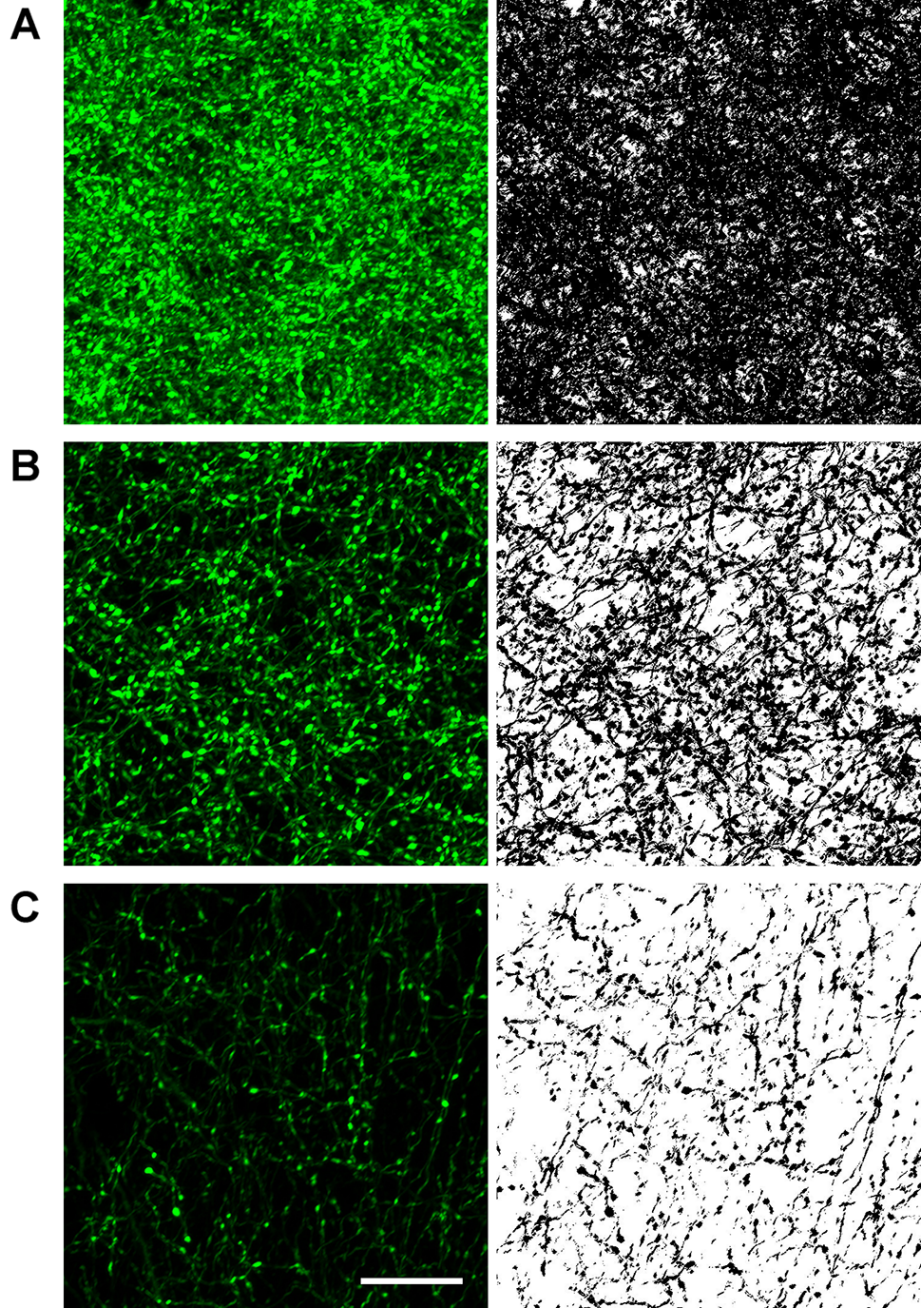
Representative examples of confocal images (right) and converted binary images (left) used to quantify fiber innervation density in a sample area (85 × 85 µm^2^). Examples are shown for the dmNAcSh (**a**), CeL (**b**), and deep layers of the infralimbic cortex (**c**). Scale bar = 20 µm

Figure 13 illustrates the ROI where confocal images were taken to carry out a quantitative analysis of the fiber density in the various regions innervated by the PVT (sample areas size = 85 × 85 µm^2^). Sampling within the ROI indicated that PVT fibers were homogenously distributed within these regions. Examples of the confocal images along with the converted binary images used for the analysis of fiber density are shown in Fig. 14. Our approach was to compare primary innervation (i.e., GFP fiber density in a ROI produced by injection of AAVrg-Cre in ROI and AAV-GFP in the PVT) versus collateral innervation (i.e., GFP fiber density in the same ROI produced by injections of AAVrg-Cre in the other areas) in separate groups of rats.

**Fig. 15:**
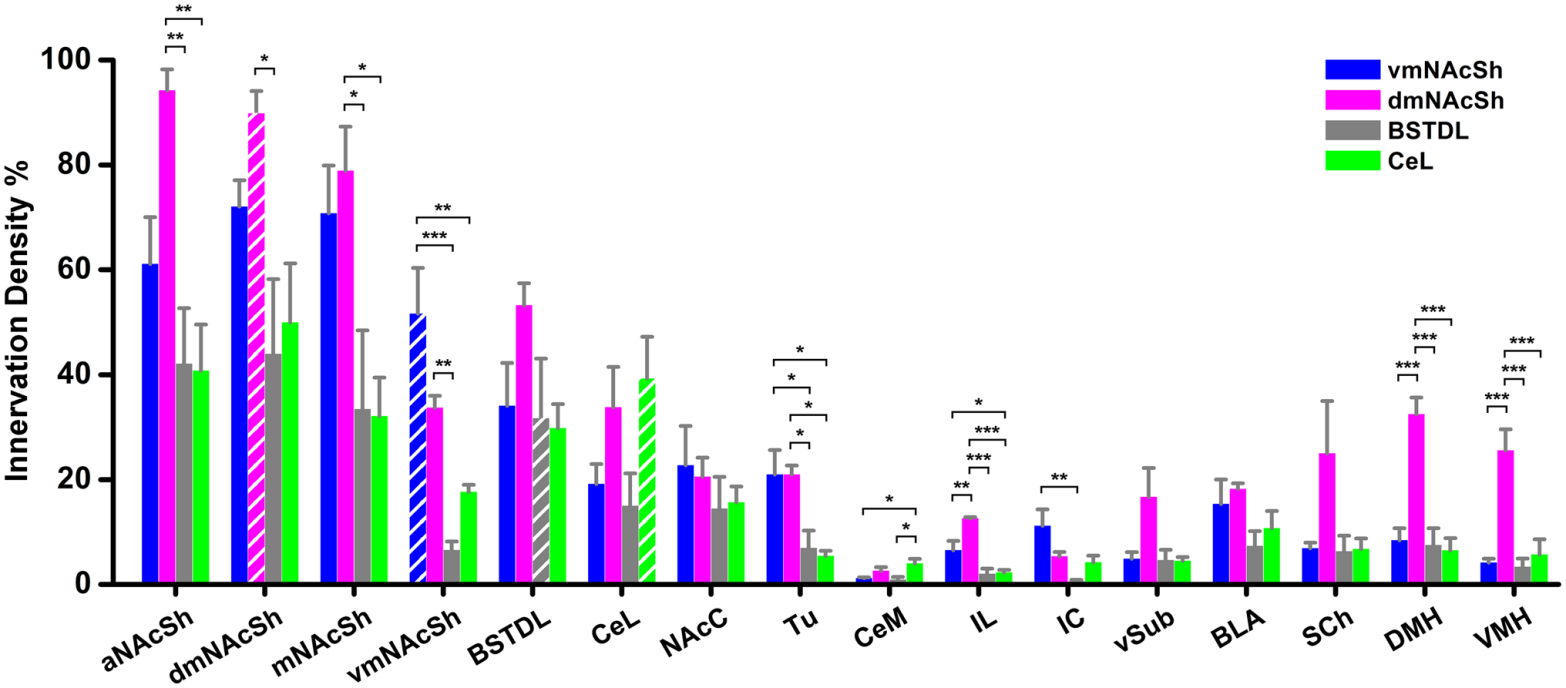
Innervation density of different areas of the brain innervated by the PVT quantified from the intersectional anterograde tracing approach. Histogram bars represent the fiber density produced in forebrain areas (horizontal axis) in cases with injections of AAVrg-Cre in the vmNAcSh, dmNAcSh, BSTDL and CeL (color coded in the graph key). Note that histogram bars with white stripes represent primary projection neurons for the area sampled (i.e., the sampled area is the same area that received the AAVrg-Cre injections) whereas solid color histogram bars represent collateral innervation from another area (i.e., area sampled is different than the area receiving the AAVrg-Cre injection). The results of the ANOVA test for the different areas sampled are as follows: aNAcSh, *F*_(3,12)_ = 8.75, *p* = 0.002; dmNAcSh, *F*_(3,12)_ = 4.74, *p* = 0.021; mNAcSh, *F*_(3,12)_ = 5.55, *p* = 0.013; vmNAcSh, *F*_(3,12)_ = 17.84, *p* < 0.001; Tu, *F*_(3,12)_ = 7.87, *p* = 0.0036; CeM, *F*_(3,12)_ = 4.62, *p* = 0.025; IL, *F*_(3,12)_ = 21.77, *p* < 0.001; IC, *F*_(3,12)_ = 6.35, *p* = 0.008; DMH, *F*_(3,12)_ = 20.07, *p* < 0.001; VMH, *F*_(3,12)_ = 16.11, *p* < 0.001; vSub, *F*_(3,12)_ = 3.93, *p* = 0.036. *n* = 4 for each group, *p < 0.05, **p < 0.01, ***p < 0.001. See list for abbreviations

Figure 15 shows the data representing the innervation density in different areas of the brain innervated by PVT quantified from the intersectional anterograde tracing experiments. For most of the ROI, the fiber density in an ROI given by the primary projection for that area produced by injections of the AAVrg-Cre in the ROI (indicated by white stripes on the color coded histogram bar) was significantly greater than the collateral projections produced by injection of the AAVrg-Cre in the other areas (color coded bars without stripes). These results offer some evidence that some PVT neurons provide a stronger projection to a ROI than what is given by collaterals originating from neurons that were identified as projecting to another ROI. This provides weak support for projection-specific neurons in the PVT (i.e., neurons with a main target). An exception is the CeL where we found that the fiber density provided by PVT-CeL projecting neurons (identified by injections of the AAVrg-Cre in the CeL) was not significantly different than the fiber density provided by collateral innervation from neurons projecting to the other ROI. It is also clear that the neurons that provide a very dense input to a ROI often provide a similar robust collateral innervation to the other ROI. For instance, cases with AAVrg-Cre injections in the dmNAcSh produced very high fiber density in the dmNAcSh and with levels of fiber density in the BSTDL and CeL comparable to the cases with AAVrg-Cre in the BSTDL and CeL. This indicates that collateral innervation of the BSTDL and CeL by PVT neurons that directly innervate the dmNAcSh is at least similar to that produced by the neurons that directly innervate the BSTDL and CeL. Conversely, it is also apparent that PVT neurons that project strongly to the BSTDL and CeL provide moderately dense collateral innervation of the NAcSh. There are a number of other notable observations. First, neurons that project preferentially to the vmNAcSh and dmNAcSh provide moderate innervation of other regions of the NAcSh and the ventral striatum including the accumbens core and olfactory tubercle. Second, neurons that primarily project to the dmNAcSh, vmNAcSh, BSTDL and CeL provide collateral innervation to the same cortical and hypothalamic regions. The density of the fiber innervation provided by collaterals to cortex and hypothalamus is relatively light to moderate compared to those to the main ROI. It is especially noteworthy that the projections to cortical areas are relatively weak and have no apparent relationship with the primary ROI targeted by the AAVrg-Cre. Third, PVT neurons that preferentially project to the dmNAcSh appear to have axons that collateralize the most with these neurons providing a uniquely dense projection to the ventral subiculum and the suprachiasmatic nucleus, dorsomedial and ventromedial nuclei of the hypothalamus.

**Fig. 16:**
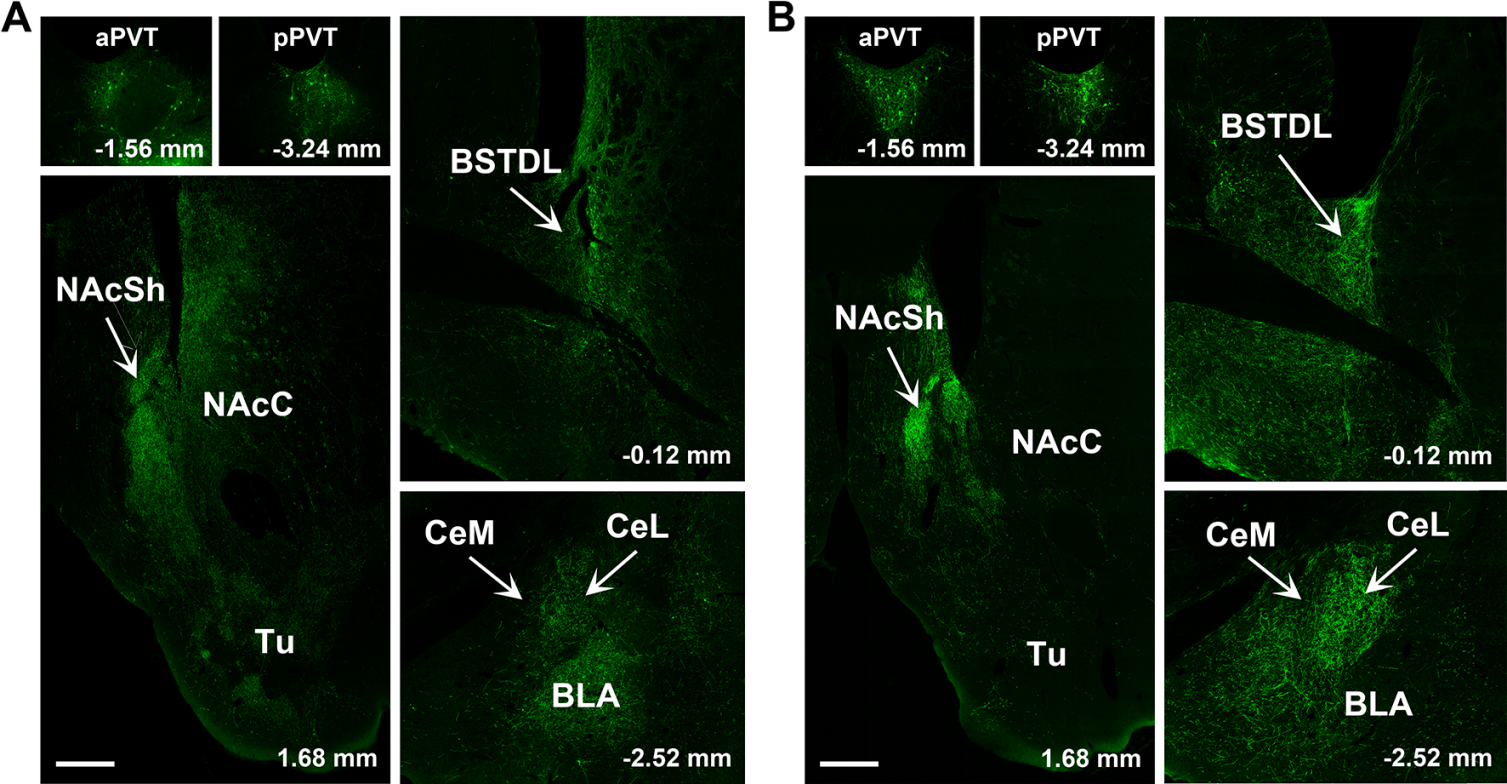
Intersectional anterograde tracing strategy applied to projection neurons in the PVT that innervate the infralimbic cortex (**a**) and dorsomedial nucleus of the hypothalamus (**b**). Note the relatively small number of PVT neurons transduced with AAVrg-Cre injections in the infralimbic cortex and dorsomedial nucleus of the hypothalamus resulted in the same pattern of labeling in the striatum, BSTDL, and CeL as seen with injections of AAVrg-Cre in the NAcSh, BSTDL and CeL. See list for abbreviations. Scale bar = 500 µm applicable to all panels. Numbers at the bottom represent distance from the bregma

We were mainly interested in the extent that PVT neurons that project primarily to the NAcSh, BSTDL and CeL provided collateral innervation to the other forebrain areas. However, the PVT also sends relatively weaker projections to a number of cortical areas and hypothalamic nuclei. Consequently, we applied the same intersectional anterograde tracing strategy to the infralimbic cortex (*n* = 2) and dorsomedial nucleus of the hypothalamus (*n* = 2) to evaluate the extent that the PVT neurons that innervate the cortex and hypothalamus provide collateral innervation to the other areas of brain (Fig. 16). Remarkably, the pattern of labeling observed was similar to the injections in the other ROI. It should be noted that injections of AAVrg-Cre in the hypothalamus or cortex resulted in fewer GFP-transduced neurons in the PVT compared to the injections of AAVrg-Cre in the other ROI. This is consistent with our previous study demonstrating that retrograde transport of traditional tracers injected in the infralimbic/prelimbic cortex to the PVT is much weaker than when tracers are injected in the NAcSh, BSTDL and CeL (Li and Kirouac 2008).

### Activation of PVT neurons by aversive conditions

**Fig. 17:**
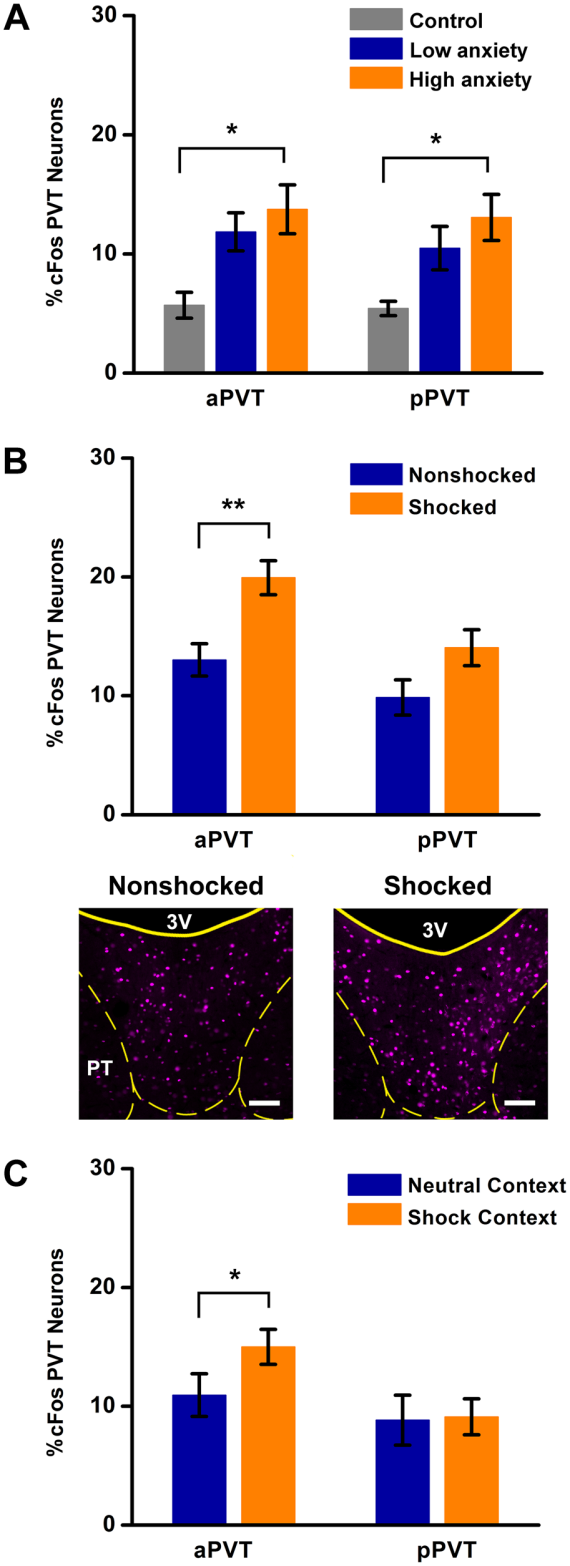
Expression of cFos in neurons of the aPVT and pPVT to aversive states. The aversive states examined included anxiety to an open field (**a**), exposure to inescapable footshocks (**b**) and retrieval of a contextual fear memory (**c**). *n* = 4 for the control, *n *= 6 for all other groups, *p < 0.05, **p < 0.01. Scale bar = 100 µm

Experiments using cFos as an indicator of neuronal activation were used to assess if the aPVT and pPVT were differentially activated in rats exposed to stressful conditions including being placed in a brightly illuminated open field, exposure to moderately intense footshocks, and retrieval of a fear memory. Overall cell counts and statistical analyses were done for the aPVT and pPVT to capture potential functional differences between subpopulation of neurons segregated in these two regions of the PVT. There was a significant difference in the percentage of the total time that rats spent in the center of the open field based on lighting condition (18.83 ± 4.40% for dim conditions vs. 3.83 ± 1.55% for brighter conditions, *F*_(1,10)_ = 12.4, *p* = 0.0055) indicating the presence of a higher anxiety state for the bright conditions. There was a significant increase in the percentage of aPVT (*F*_(2,13)_ = 5.86, *p* = 0.015) and pPVT (*F*_(2,13)_ = 5.11, *p* = 0.023) neurons expressing cFos in the high anxiety group (Fig. 17a). Exposure of rats to footshocks resulted in an increase in the percentage of PVT neurons expressing cFos in the aPVT (Fig. 17b, *F*_(1,10)_ = 10.99, *p* = 0.008). Rats exposure to the shock-associated context displayed significantly more freezing (84.4 ± 6.4% of the total time) than rats exposed to the neutral context (1.1 ± 1.1%, *F*_(1,10)_ = 164.88, *p* < 0.001). Retrieval of the contextual fear memory increased the percentage of aPVT neurons expressing cFos (Fig. 17c, *F*_(1,10)_ = 7.83, *p* = 0.019). There were no significant differences in the percentage of pPVT neurons that expressed cFos to footshock exposure or retrieval of the contextual fear memory.

**Fig. 18:**
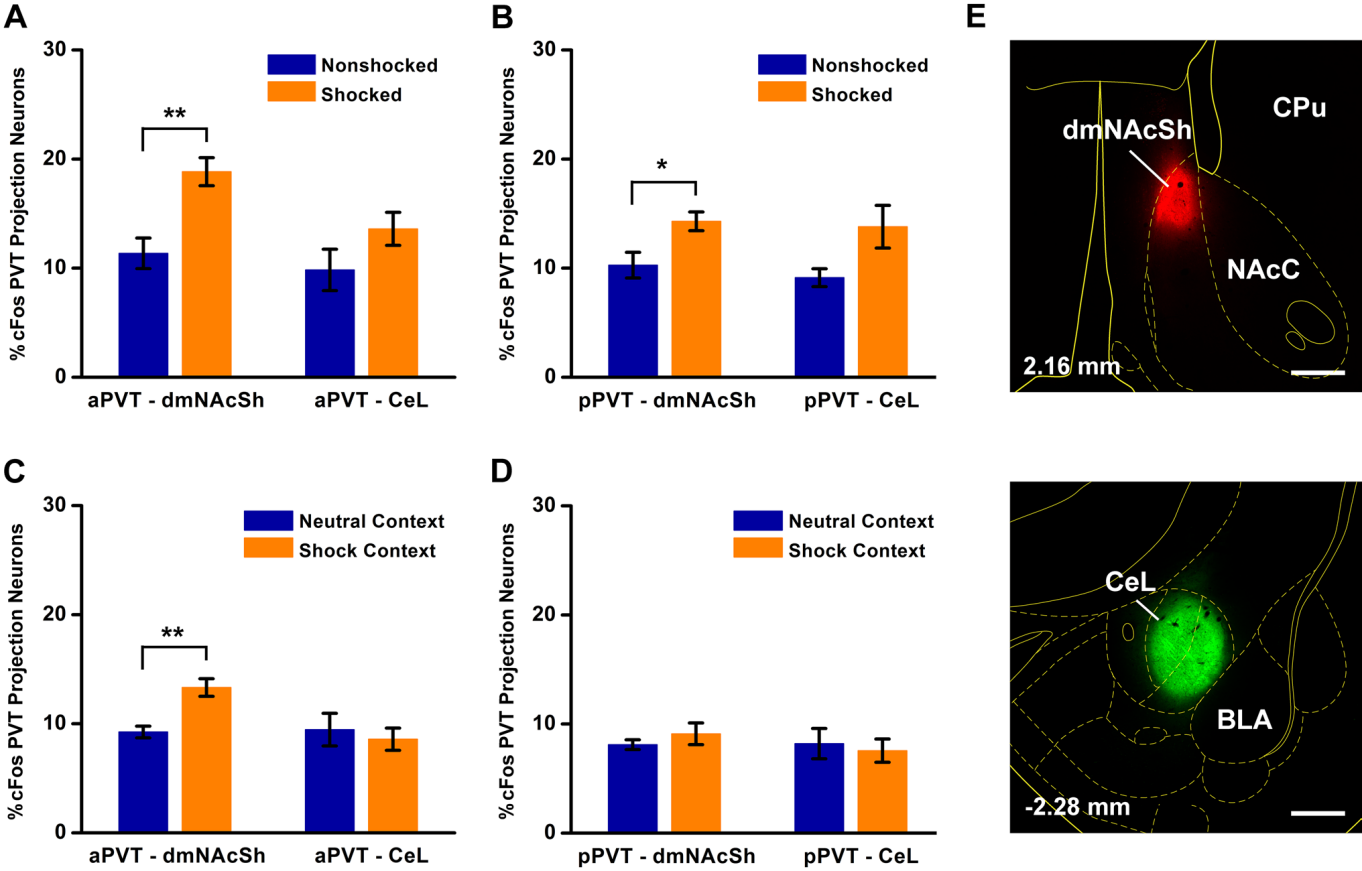
Expression of cFos in projection-specific PVT neurons from exposure to footshocks and retrieval of a contextual fear memory. The figure displays the expression of cFos in projection-specific neurons in the aPVT and pPVT to footshock exposure (**a**, **b**) and retrieval of a contextual fear memory (**c**, **d**) with examples of the size and location of the CTB injections in the dmNAcSh and CeL used for the experiments (**e**). *n* = 6 for each group, *p < 0.05, **p < 0.01. Numbers at the bottom represent distance from the bregma. Scale bar = 500 µm. See list for abbreviations

Rats that were used to assess if PVT neurons were activated by footshocks or retrieval of a contextual fear memory had received injections of CTB in the CeL and the dmNAcSh (combination injections in the same animal). We were specifically interested in whether PVT neurons that project to the CeL, an area well known for its involvement in fear (LeDoux et al. 1988), were differentially activated compared to neurons that project to the dmNAcSh, a region of the NAcSh most closely linked to appetitive responses (Berridge and Kringelbach 2015). For footshock exposure, there was a significant increase in cFos expressing neurons in the aPVT (Fig. 18a, *F*_(1,10)_ = 15.31, *p* = 0.003) and pPVT (Fig. 18b, *F*_(1,10)_ = 7.59, *p* = 0.02) that projected to the dmNAcSh. Lastly, there was a significant increase in the percentage of aPVT neurons projecting to the dmNAcSh expressing cFos to retrieval of the contextual fear memory (Fig. 18c, *F*_(1,10)_ = 17.84, *p* = 0.002) but no apparent activation of aPVT neurons projecting to the CeL or pPVT neurons projecting to either the dmNAcSh or CeL (Fig. 18c, d).

## Discussion

The PVT sends dense fiber projections to the NAcSh, BSTDL, IPAC and CeL (Li and Kirouac 2008; Vertes and Hoover 2008; Moga et al. 1995), brain regions that form an anatomical macrostructure referred to as the extended amygdala (Alheid et al. 1995; de Olmos et al. 2004; Heimer and Alheid 1991). In addition to these projections, the PVT provides considerably less dense projections to limbic cortical areas and the hypothalamus. The PVT also weakly innervates a number of brain regions immediately adjacent to those mentioned above. The results of the triple-injection experiments reported here are consistent with our previous study involving various combinations of the CTB injections where we found that many neurons in the PVT provide collateral innervation of the dmNAcSh, vmNAcSh, BSTDL and CeL (Dong et al. 2017). It is clear from our previous and the present study that neurons that provide collateral innervation are found in the aPVT and pPVT as intermixed populations without forming distinct clusters. However, it is important to understand that retrograde tracers provide an incomplete picture of the innervation provided by a population of neurons because neurons can bifurcate extensively to give off fiber terminals (Rodriguez-Lopez et al. 2017; Prensa et al. 2009; Prensa and Parent 2001). It is also impossible to know how many fibers and terminals are provided by a population of neurons labeled from injections of retrograde tracer in a terminal field. For this reason, we applied an intersectional anterograde tracing strategy to thoroughly evaluate the extent that PVT neurons project to one region of the forebrain provide collateral innervation to other regions. While we did expect that PVT neurons would provide collaterals to some of its main targets of innervation, the extent of this fiber divergence was rather astounding. The anterograde experiments indicate that most PVT neurons provide axons that bifurcate extensively to innervate multiple regions of the forebrain. Furthermore, quantitative analysis of projection specific versus collateral innervation of the NAcSh, BSTDL and CeL provides little support for the view that some PVT neurons project specifically to one of these regions at the exclusion of others. The results of the tracing experiments presented here and previously (Dong et al. 2017) offer compelling evidence that most neurons in the PVT project to the NAcSh and that these neurons bifurcate extensively to give projections to many other regions of the forebrain. Experiments using cFos as a measure of neuronal activation also show that neurons in both the aPVT and pPVT are activated by aversive events. A population of aPVT-dmNAcSh projecting neurons that forms an especially robustly divergent projection system was found to be distinctively activated by aversive events. These findings are consistent with the view that neurons in the PVT are responsive to stress and other homoeostatic challenges and that the PVT promotes adaptive behavioral responses by acting on a number of forebrain regions (Hsu et al. 2014; Kirouac 2015; Millan et al. 2017; Barson et al. 2020; McGinty and Otis 2020). The divergent nature of PVT neurons suggests that this thalamic nucleus coordinates adaptive behavioral, physiological and neuroendocrine responses to stress.

### Methodological considerations

While the extent that PVT neurons send axonal collaterals to different regions of the brain can be examined using multi-label retrograde tracing, this approach is likely to yield an underestimation in how much a population of neurons provides collateral innervation (Schofield et al. 2007). As shown for the ascending dopamine neurons, the amount of fiber terminals provided by a neuron to the striatum and cortex is impossible to estimate from the number of labeled neurons produced by injections of a retrograde tracer in these terminal field areas (Rodriguez-Lopez et al. 2017; Prensa et al. 2009; Prensa and Parent 2001). Meticulous tracing of single neurons exquisitely demonstrates the axonal trajectories and fiber terminals of neurons in a way that cannot be shown using other methods. However, the work is painstaking and can only assess a handful of neurons from a large population (Rodriguez-Lopez et al. 2017; Tripathi et al. 2010, 2013; Aransay et al. 2015; Prensa et al. 2009).

The intersectional anterograde tracing approach was developed by our group to study the extent that a large population of PVT neurons provide collateral innervation to its forebrain targets. The approach is unique in that it combines retrograde and anterograde tracing methods in the same experiment in a way that is useful for studying how axons from a large population of neurons bifurcate to innervate other regions. The approach was used in the present study to evaluate the extent that projection-specific neurons in the PVT provide collaterals to other areas of the forebrain in addition to its projection-specific target (i.e., comparison of fiber density in a ROI produced by injecting AAVrg-Cre in the target area vs. other areas innervated by the PVT). However, it should be understood that what we call projection-specific neurons are neurons with axons that bifurcate multiple times to innervate a number of regions in addition to the ROI targeted. The usefulness of the approach requires that the AAVs are able to reliably transduce neurons in both the retrograde and anterograde directions in patterns that are consistent with what have been reported using traditional and non-viral tracing substances. For instance, the approach resulted in more neurons transduced in the aPVT when AAVrg-Cre was injected in the dmNAcSh and more neurons were transduced in the pPVT when AAVrg-Cre was injected in CeL. Importantly, the method resulted in neurons being transduced primarily in the PVT and not in other thalamic nuclei. The pattern of anterograde labeling that was observed using this method was similar to what has been reported using non-viral anterograde tracers (Li and Kirouac 2008; Parsons et al. 2007; Vertes and Hoover 2008).

Anterograde labeling produced from specifically targeting the aPVT and pPVT using the intersectional approach was not carried out because of previous findings that neurons innervating the different areas of the extended amygdala originate from intermixed populations in both regions of the PVT (Dong et al. 2017). The purpose of the intersectional anterograde experiments was to compare the projection patterns of PVT neurons that innervate different subcortical targets via primary and collateral projections. While some anatomical differences have been noted between aPVT and pPVT, the distinction is largely arbitrary and not based on clear anatomical reasons (Kirouac 2015; Barson et al. 2020; McGinty and Otis 2020).

As a final point, it is impossible based on combination injections of CTB to absolutely discount the possibility that a subpopulation of topographically organized neurons in the PVT project to a subregion of the extended amygdala. The extended amygdala occupies a large and variable region of the basal forebrain and the CTB injections only involved a portion of the regions investigated. This has been partly mitigated by making injections of CTB in regions known to receive the densest PVT innervation. As discussed below, the common pattern of anterograde labeling observed across the extended amygdala produced by the intersectional approach also suggests that such subpopulations are unlikely to exist.

### Extensive divergence of PVT projections

The results of the anatomical tracing experiments reported here are in line with what has been previously documented using both retrograde and anterograde tracing methods (Dong et al. 2017; Li and Kirouac 2008; Vertes and Hoover 2008). It is clear that the NAcSh, BSTDL and CeL are the areas of the brain most densely innervated by the PVT and that neurons that project to these regions do not form distinct clusters of uniquely localized cells in the PVT. In addition, neurons that project to the dmNAcSh are primarily found in the aPVT while those to the vmNAcSh are primarily found in the pPVT, albeit as intermixed populations (Dong et al. 2017). A number of other areas of the forebrain are also innervated by the PVT (Li and Kirouac 2008; Vertes and Hoover 2008; Moga et al. 1995) but the density of these projections is orders of magnitude weaker than those to the NAcSh, BSTDL, and CeL. Here we provide an estimation of the axon density in brain regions innervated by the PVT based on optical confocal imaging of small areas (85 × 85 µm^2^ area averaged over four similar cases). It should be understood that the anterograde analysis of fiber projections to the different regions examined was not unbiased in that the primary areas sampled (i.e., NAcSh, BSTDL, and CeL) were those containing a high density of fibers while other areas sampled contained moderate to weak fiber density. Based on the fact that the number and size of the areas sampled in the brain were limited, the data should be considered as estimations and interpreted cautiously. Many of the weakly innervated areas were not quantified in the present study but we would expect that all areas innervated by the PVT would receive some level of collateral innervation (as visually evident in Figs. 9, 10, 11, 12). For example, it is clear that the dorsal striatum, medial regions of the bed nucleus of the stria terminalis, as well as the cortical and basomedial nucleus of the amygdala received collateral innervation. Previous anterograde tracing studies have not been in total agreement in term of strength of the PVT projection to infralimbic and prelimbic cortex (Li and Kirouac 2008; Vertes and Hoover 2008; Moga et al. 1995). The present investigation is consistent with our previous anterograde tracing study (Li and Kirouac 2008) where we found a weak PVT innervation of the infralimbic-prelimbic cortex. However, it should be understood that regardless of the strength of the PVT projection to the prefrontal cortex, this projection is functional at the synaptic level (Huang et al. 2006; Matyas et al. 2018).

Most neurons in the PVT project to the NAcSh (Dong et al. 2017) and we conclude from the results of the intersectional tracing experiments that the axons of many of the NAcSh projecting neurons send collaterals to other regions of the forebrain. It is also clear from these experiments that neurons that primarily project to the BSTDL and CeL also send collaterals to many regions of the forebrain including the NAcSh. Nonetheless, it is impossible to know from these experiments whether the neurons identified as primarily projecting to the BSTDL or CeL are unique projection neurons different from those neurons that project to the NAcSh. Based on the overall pattern of fiber labeling observed from the intersectional anterograde tracing experiments and the fact that most neurons in PVT project to the NAcSh (Dong et al. 2017), we postulate that the PVT fibers to the BSTDL and CeL are collaterals issued from PVT neurons that innervate the NAcSh. This conclusion is supported by single-cell tracing experiments where the main axon of a PVT neurons was seen to give collaterals that terminated in some of the same areas identified here (Matyas et al. 2018; Unzai et al. 2015). However, it is possible that there are subpopulations of neurons with a primary target of innervation that also provide collateral fibers to a unique combination of other regions in the forebrain.

As discussed above, PVT neurons also provide some fiber projections to a number of cortical areas and hypothalamic nuclei. Application of the intersectional anterograde tracing strategy to the infralimbic cortex and dorsomedial nucleus of the hypothalamus produced the same pattern of labeling in the NAcSh, BSTDL and CeL, albeit much less dense than cases involving injections of the AAVrg-Cre in the extended amygdala. Accordingly, it is likely that the labeling observed in the hypothalamus or infralimbic cortex with injections in the NAcSh, BSTDL and CeL originated mainly from collaterals issued by PVT neurons innervating these regions and not neurons that primarily innervate the cortex or hypothalamus. As previously reported there is a high likelihood of antidromic-orthodromic activation of axon collaterals when stimulating PVT fibers optogenetically (Matyas et al. 2018). Consequently, the behavioral effects of stimulating PVT fibers in any forebrain region could be mediated by a number of regions in addition to the one being targeted.

### Activation of PVT neurons by aversive events

The PVT is an area of the brain that is often reported to be activated by aversive events including restraint (Bhatnagar and Dallman 1998; Cullinan et al. 1995); tail pinch and footshocks (Bubser and Deutch 1999; Smith et al. 1997; Baisley et al. 2011; Yasoshima et al. 2007); swimming stress (Cullinan et al. 1995; Zhu et al. 2011); predator scent (Baisley et al. 2011); ultrasonic vocalizations in the dysphoric range (Beckett et al. 1997); aversive visceral stimulation (Xu and Sudhof 2013; Yasoshima et al. 2007); food deprivation (Timofeeva and Richard 2001); omission of an expected reward (Do-Monte et al. 2017); and withdrawal from addictive drugs (Zhou and Zhu 2019; Barson et al. 2020; Kirouac 2015; Millan et al. 2017). While some studies have noted differential activation of the cfos gene in the aPVT and pPVT with the pPVT sometimes highlighted as being more robustly activated, many studies have not done or reported regional comparisons in a way that allows for unequivocal conclusions to be reached. The present study examined cFos expression in the aPVT and pPVT to aversive conditions in addition to the expression of cFos relative to neurons that project primarily to dmNAcSh and CeL. We found that neurons in the aPVT were activated by different aversive events while neurons in the pPVT were not. We also observed that aPVT-dmNAcSh projecting neurons were activated whereas aPVT- and pPVT-CeL projecting neurons were not when rats were exposed to an anxiogenic open field, footshocks, and retrieval of a contextual fear memory. These findings stand in contrast to another study in mice that reported that neurons in the pPVT that express dopamine D2 receptors were activated by an aversive event while neurons in the aPVT that lack these receptors were not (Gao et al. 2020). However, comparison between these two studies is difficult considering that the experiments were done in different species and different indictors of neuronal activation were used (i.e., cFos vs. calcium imaging).

PVT neurons have been shown to be responsive to appetitive conditions and the cues that predict their occurrences (for more details see reviews by Zhou and Zhu 2019; Barson et al. 2020; Kirouac 2015, 2021; Millan et al. 2017; McGinty and Otis 2020). While a number of studies have employed single unit recording or calcium imaging to examine if PVT neurons respond to appetitive and aversive stimuli (reviewed in McGinty and Otis 2020; Barson et al. 2020), most studies have not made comparisons between the aPVT and pPVT or whether the same neuron responds to both emotional valences. Recent studies in awake animals have reported that PVT neurons show an increase in activity when animals are exposed to novel appetitive and aversive stimuli (Choi et al. 2019; Zhu et al. 2018). These responses are graded relative to the emotional intensity of the stimuli and occur when conditioned cues are presented (Choi et al. 2019; Zhu et al. 2018). Single unit recordings of PVT neurons located at approximately a mid-PVT level demonstrated that many units responded in a valence-independent manner and that the activity of these neurons was enhanced in a graded manner to more aversive unconditioned stimuli (Zhu et al. 2018). It was also reported that the calcium signal was enhanced in the aPVT in the presence of cues that predicted the aversive event during a motivational conflict situation when the cues predicted a sucrose reward (Choi et al. 2019). We can surmise from these recent studies that PVT neurons respond to and track the saliency of emotionally relevant stimuli and that these neurons may be particularly tuned to aversive stimuli. Furthermore, single unit recording and calcium imagining of neurons in the PVT indicate that PVT neurons are generally active during periods of behavioral arousal and are especially robustly activated by aversive states (Ren et al. 2018; Matyas et al. 2018). It is possible that certain aversive events (e.g., physiological vs. psychological) or in the presence of particular conditions (e.g., presence of a motivational conflict or other behavior states) may result in differential activation of neurons in the aPVT or pPVT and/or a subset of PVT neurons with different projection targets. Indeed, activation of different divergent projecting pathways may have complex behavioral effects as elegantly demonstrated in recent studies (Choi et al. 2019; Do-Monte et al. 2017).

### Functional considerations

Axon collateralization is an important characteristic of many projection pathways (Rockland 2018) including the ascending mesostriatal dopamine and the descending ventral striatopallidal systems involved in regulating behavior (Rodriguez-Lopez et al. 2017; Tripathi et al. 2010, 2013; Aransay et al. 2015; Prensa et al. 2009). Collaterals allow the same information to be sent to multiple regions in a way that permits a group of neurons to coordinate activity in multiple areas of the brain (Rockland 2018). For example, compelling evidence has been provided that the PVT and other neurons of the dorsal midline thalamus regulate arousal states and sleep through a projection to the ventral striatum (Ren et al. 2018; Matyas et al. 2018). It is possible that groups of divergently projecting PVT neurons could modulate behavioral states and arousal by synchronously modulating a number of forebrain regions. Previous investigations have provided evidence that projection-specific neurons in the PVT integrate signals related to emotionally salient cues and behavioral state, and then feed-forward signals to specialized downstream circuits to elicit an appropriate behavioral response (Zhang and van den Pol 2017; Penzo et al. 2015; Do-Monte et al. 2015, 2017; Otis et al. 2019; Choi et al. 2019; Dong et al. 2020). The PVT has been implicated in a wide range of behavioral responses including food intake, reward and drug seeking, fear, anxiety, and stress-related responses. How divergent projection from neurons in the PVT modulate such diverse and sometimes incompatible behaviors is difficult to fully understand. Details of the evidence implicating the PVT in motivated behavior along with some of the challenges of studying this nucleus are discussed in a recent review (McGinty and Otis 2020).

**Fig. 19:**
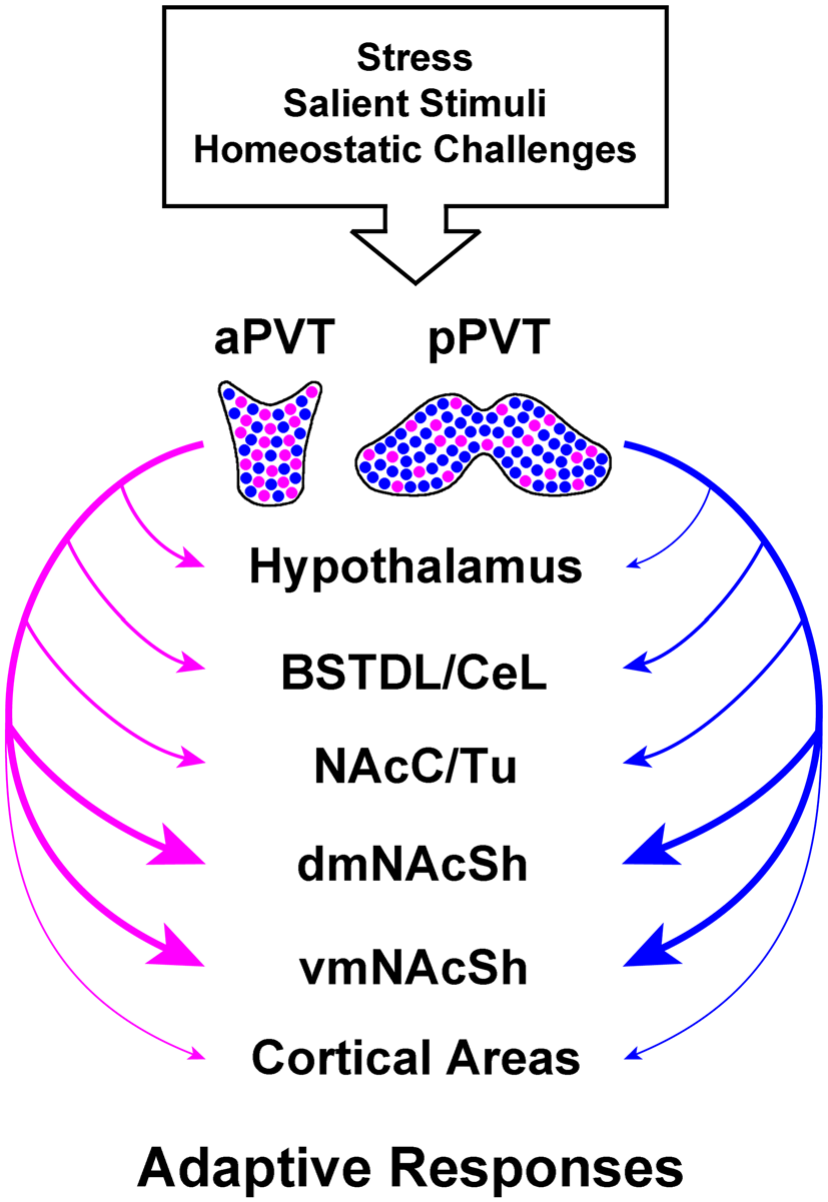
Summary of the collateral projections associated with neurons in the PVT that project to the dmNAcSh and vmNAcSh based on the retrograde experiments from this study and our previous paper (Dong et al. 2017) in addition to the intersectional anterograde tracing experiments. The size of the arrows indicates the strength of the projection as estimated by the innervation density from the anterograde tracing experiments. Note that these two population of neurons are located along the anterior–posterior extent of the PVT as intermixed populations. See list for abbreviations

It is common to consider the aPVT and pPVT as distinct regions based on the anatomical differences between these two regions (Vertes and Hoover 2008; Vertes et al. 2015; Li and Kirouac 2008, 2012). Figure 19 illustrates an updated view of how the PVT is organized based on the results of the present investigation and our previous study using combinations of CTB injections (Dong et al. 2017). We propose that it may be more appropriate to view individual groups of neuron with common sets of divergent projections as the functional unit for the PVT. We postulate that activation or inhibition of neurons that innervate combinations of forebrain areas may result in well-coordinated behavioral, physiological and neuroendocrine responses. The highly divergent neurons identified as innervating the dmNAcSh is a case in point. These neurons not only project densely to the dmNAcSh, but also provide strong collateral innervation to the BSTDL, CeL, ventral subiculum, hypothalamus and many other areas of the brain. Since these neurons appear to be activated by aversive conditions, they may be important for generating a coordinated response to a stressful event by simultaneously acting on multiple regions of the forebrain. We can make general predictions of the types of responses that could be mediated by these dmNAcSh projecting neurons based on what we know about the PVT and the areas innervated by these neurons. First, activation of fibers to the dmNAcSh could lead to inhibition of appetitive responses (Berridge 1996; Otis et al. 2019; Do-Monte et al. 2017; Dong et al. 2020) which are incompatible with a threatening situation. Second, activation of fibers in the BSTDL and CeL could promote behavioral responses related to fear and anxiety (Penzo et al. 2015; Do-Monte et al. 2015). Third, activation of the fibers to the ventral subiculum, dorsomedial nucleus of the hypothalamus could help modulate the physiological and hormonal responses to the threat including circadian and biological rhythms (Herman et al. 2016; Herman and Mueller 2006; DiMicco et al. 2002; Colavito et al. 2015; Saper et al. 2005; Bhatnagar and Dallman 1998, 1999).

Other groups of neurons with different combinations of collateral innervation targets may be tuned to appetitive stimuli and exert influence via different combination of innervation targets. It is not surprising that global PVT manipulations have been shown to produce mixed and inconsistent behavioral outcomes (McGinty and Otis 2020) considering the divergent nature of PVT neurons. Global manipulation of either the aPVT or pPVT is likely to result in interference or activation of a mixed population of neurons with complex divergent projections. A useful approach to investigate the PVT may be to study specific neuron subtypes that form a distinctive divergent projection system. It may be also possible to target uniquely divergent PVT neurons that have distinctive receptors or functional neuropeptides (McGinty and Otis 2020; Barson et al. 2020; Gao et al. 2020).

The literature is rich with examples of how the PVT is activated by aversive stress (Hsu et al. 2014; Kirouac 2015) and its role in modulating the hormonal and behavioral effects of both acute and chronic stress (Dong et al. 2020; Li et al. 2010; Pliota et al. 2018; Bhatnagar and Dallman 1998, 1999; Bhatnagar et al. 2000, 2002, 2003; Jaferi and Bhatnagar 2006; Jaferi et al. 2003; Heydendael et al. 2011). While the term stress is often used to refer to challenges involving strong negative emotions (i.e., aversive stress), recent definitions emphasize that stress also includes homoeostatic challenges like the physiological demands of thirst, hunger and disruptions in circadian rhythms (Goldstein and McEwen 2002; Karatsoreos and McEwen 2011; McEwen and Akil 2020). The PVT is a region of the thalamus that is active during states of high arousal and following challenging conditions. State- and saliency-responsive neurons in the PVT potentially represent a powerful system for coordinating a wide range of adaptive responses needed to achieve stability of homeostatic systems in a process that has been termed allostasis (Goldstein and McEwen 2002; Karatsoreos and McEwen 2011; McEwen and Akil 2020).

## Data Availability

Available with reasonable request.

## Abbreviations

3V: Third ventricle
ac: Anterior commissure
AM: Anteromedial nucleus of thalamus
BLA: Basolateral amygdala
BLP: Basolateral amygdala, posterior
BSTDL: Bed nucleus of stria terminalis, dorsolateral
BSTDM: Bed nucleus of stria terminalis, dorsomedial
CeL: Central amygdala, lateral-capsular division
CeM: Central amygdala, medial
CM: Central medial nucleus of thalamus
CPu: Caudate putamen
CTB: Cholera toxin subunit B
DMH: Dorsomedial hypothalamus
DP: Dorsal peduncular cortex
fmi: Forceps minor corpus callosum
fr: Fasciculus retroflexus
Hb: Habenular nucleus
ic: Internal capsule
IC: Insular cortex
IL: Infralimbic cortex
IMD: Intermediodorsal nucleus of thalamus
IPAC: Interstitial nucleus of the posterior limb of the anterior commissure
LV: Lateral ventricle
MD: Mediodorsal nucleus of thalamus
NAc: Nucleus accumbens
NAcC: Core of the nucleus accumbens
aNAcSh: Anterior aspect of the shell of the nucleus accumbens
dmNAcSh: Dorsomedial region of the shell of the nucleus accumbens
mNAcSh: Middle aspect of the shell of the nucleus accumbens
pNAcSh: Posterior aspect of the shell of the nucleus accumbens
vmNAcSh: Ventromedial region of the shell of the nucleus accumbens
opt: Optic tract
PrL: Prelimbic cortex
PT: Paratenial nucleus of thalamus
PVT: Paraventricular nucleus of the thalamus
aPVT: Anterior aspect of the paraventricular nucleus of the thalamus
pPVT: Posterior aspect of the paraventricular nucleus of the thalamus
SCh: Suprachiasmatic nucleus
ROI: Region of interest
Rt: Reticular nucleus of the thalamus
St: Stria terminalis
Tu: Olfactory tubercle
VMH: Ventromedial hypothalamus
VP: Ventral pallidum
vSub: Ventral subiculum
